# Pathophysiological and molecular mechanisms involved in renal congestion in a novel rat model

**DOI:** 10.1038/s41598-018-35162-4

**Published:** 2018-11-14

**Authors:** Satoshi Shimada, Takuo Hirose, Chika Takahashi, Emiko Sato, Satoshi Kinugasa, Yusuke Ohsaki, Kiyomi Kisu, Hiroshi Sato, Sadayoshi Ito, Takefumi Mori

**Affiliations:** 1 Division of Nephrology, Endocrinology and Vascular Medicine, Tohoku University Graduate School of Medicine, Sendai, Japan; 2 Division of Nephrology and Endocrinology, Tohoku Medical and Pharmaceutical University, Sendai, Japan; 3 Division of Clinical Pharmacology and Therapeutics, Tohoku University Graduate School of Pharmaceutical Sciences, Sendai, Japan; 4 Division of Integrative Renal Replacement Therapy, Tohoku University Graduate School of Medicine, Sendai, Japan

## Abstract

Increased central venous pressure in congestive heart failure causes renal dysfunction; however, the underlying mechanisms are unclear. We created a rat renal congestion model and investigated the effect of renal congestion on hemodynamics and molecular mechanisms. The inferior vena cava (IVC) between the renal veins was ligated by suture in male Sprague-Dawley rats to increase upstream IVC pressure and induce congestion in the left kidney only. Left kidney congestion reduced renal blood flow, glomerular filtration rate, and increased renal interstitial hydrostatic pressure. Tubulointerstitial and glomerular injury and medullary thick ascending limb hypoxia were observed only in the congestive kidneys. Molecules related to extracellular matrix expansion, tubular injury, and focal adhesion were upregulated in microarray analysis. Renal decapsulation ameliorated the tubulointerstitial injury. Electron microscopy captured pericyte detachment in the congestive kidneys. Transgelin and platelet-derived growth factor receptors, as indicators of pericyte-myofibroblast transition, were upregulated in the pericytes and the adjacent interstitium. With the compression of the peritubular capillaries and tubules, hypoxia and physical stress induce pericyte detachment, which could result in extracellular matrix expansion and tubular injury in renal congestion.

## Introduction

The physiological association between the kidney and heart has received significant attention. Reduction of cardiac output induces renal hypoperfusion, which causes renal ischemia. On the other hand, renal venous hypertension leads to high renal interstitial hydrostatic pressure (RIHP) and reduction in glomerular filtration rate (GFR)^1^. In patients with chronic congestive heart failure (CHF), central venous pressure (CVP) normally measured at the inferior vena cava (IVC) and renal venous pressure (RVP) are higher than in healthy subjects^2^. Mullens *et al*. demonstrated that CVP, rather than cardiac index, correlates with GFR in patients with advanced decompensated CHF^3^. Damman *et al*. reported that CVP is associated with GFR, and is a strong predictor of mortality^4^. Thus, CVP is an indicator of both renal dysfunction and CHF. However, the underlying mechanisms of renal dysfunction in patients with CHF, are yet unknown.

Increased CVP raises RVP, causing renal congestion and renal dysfunction^5,6^. Several animal studies have demonstrated that acute increases in RVP from renal vein constriction increases RIHP, decreases renal blood flow (RBF), and results in sodium retention and diuresis^7–9^. Ligation of the IVC above the renal veins also raises RVP^10^ and induces chronic renal dysfunction^11,12^.

Rat models in which RIHP is elevated, such as ureteral obstruction^13^ and aortic stenosis^14,15^ cause renal injury. Fibrogenesis via epithelial-mesenchymal transition is the main causative factor of renal injury in these models^15,16^. Recently, studies on pericyte-myofibroblast transition have led to novel theories of kidney fibrosis^17^. Other pathways are involved in tissue fibrosis^18^; however, little is known about the molecular mechanisms in renal congestion and their roles in CHF-related renal injury. We hypothesized that RIHP and vasa recta expansion pressures are responsible for renal congestion-mediated fibrosis. Thus, we created a novel rat renal congestion model and investigated the effect of renal congestion on hemodynamics and its molecular mechanisms. Most previous studies have been conducted in acute phases of renal congestion, and few have focused on molecular mechanisms underlying its pathophysiology. Therefore, we aimed to determine the pathogenetic mechanism of renal injury on renal congestion.

We created a rat renal congestion model by ligating the IVC between the renal veins, which allowed us to compare congestive left kidneys (congestive kidneys) to non-congestive right kidneys (control kidneys). To determine the molecules underlying renal congestion pathophysiology, gene expression arrays of the left and right kidneys were compared within subjects. The functional and structural characteristics of the kidneys were examined using immunohistological analysis and electron microscopy.

## Results

### Acute experiments to evaluate renal congestion effects on renal hemodynamics

**Figure 1:**
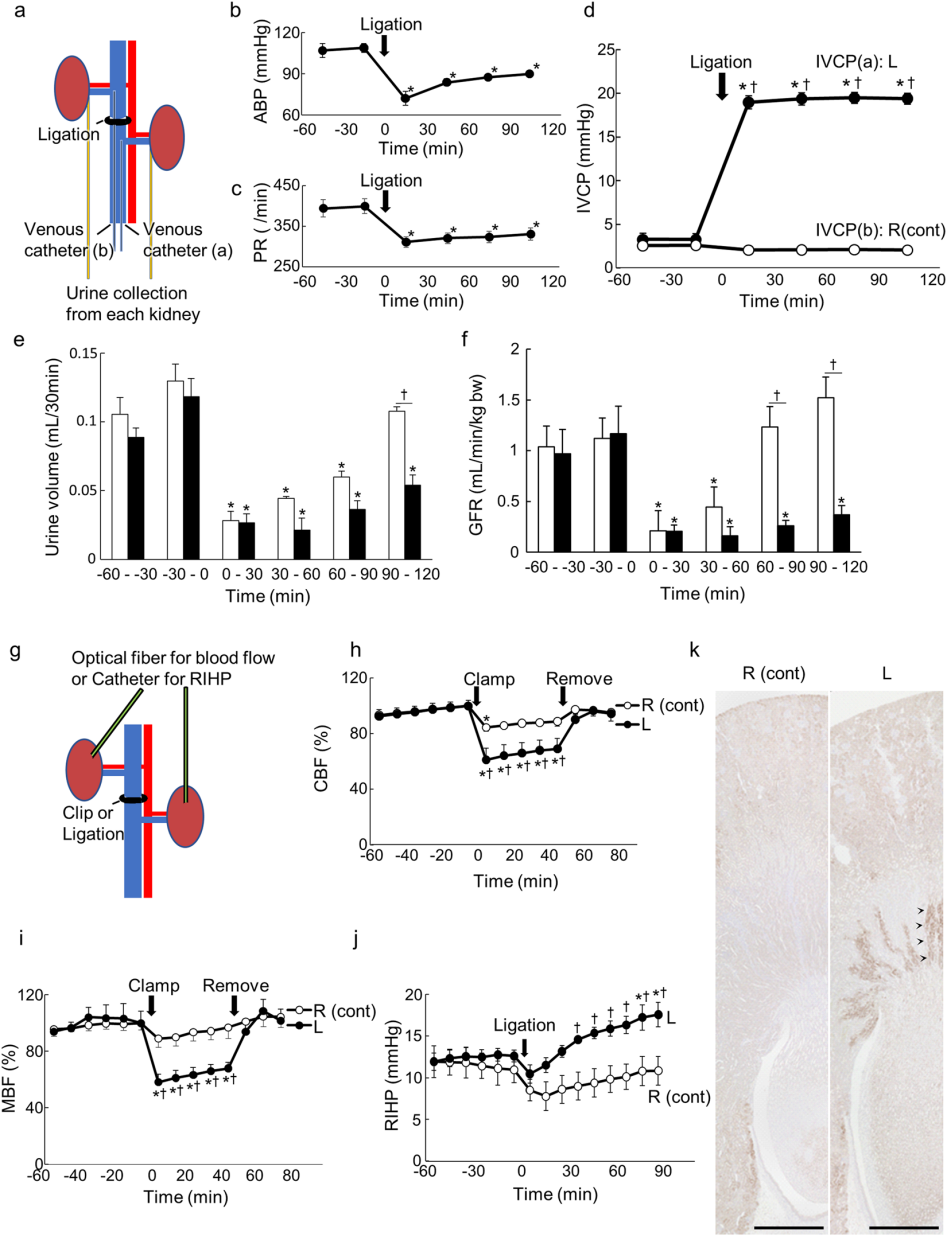
Acute animal experiments. (**a**) The schema of the acute animal experiments for GFR and IVCP. The catheters were detained for measurement of IVCP and urine collection from each kidney. Ligation was performed at time 0 after the 60-min baseline. ABP (**b**), PR (**c**), IVCP (**d**), urine volume (**e**), and GFR (**f**) were measured at the same time of each experiment. n = 5, ▫ indicates the control kidneys, ▪ indicates the congestive kidneys. (**g**) The schema of the blood flow or RIHP experiment. Optical fibers were inserted into the both renal cortical or outer medullary regions for measurement of CBF and MBF. For RIHP measurement, catheters were placed in the cortex. Clamp or ligation was performed at time 0 after the 60-min baseline. The clamp was released after 50 min. CBF n = 4 (**h**), MBF n = 5 (**i**), and RIHP n = 4 (**j**) were measured separately. ◦ indicates control kidneys, ● indicates congestive kidneys. Data are presented as the means ± SEM of a 10-min average. *p < 0.05 versus baseline (−10 to 0 min), ^†^p < 0.05 versus right; Tukey’s test. (**k**) Renal hypoxia analysis by pimonidazole staining in the acute phase. Scale bar, 1.0 mm. Allow heads show the pimonidazole positive areas. ABP: arterial blood pressure; PR: pulse rate; IVCP: inferior vena cava pressure; R (cont): control right kidney; L: congestive left kidney; CBF: cortical blood flow; MBF: medullary blood flow; RIHP: renal interstitial hydrostatic pressure.

The catheters were detained for measurement of IVC pressure (IVCP) and urine collection from each kidney. IVC between the renal veins was ligated after the 60-min baseline (Fig. 1a). arterial blood pressure (ABP) and pulse rate (PR) were immediately decreased after ligation. Although there was a tendency for these to gradually improve, ABP and PR did not return to baseline within 120 min (Fig. 1b,c). Upstream IVCP increased to around 20 mmHg immediately after ligation and remained at that level throughout the experiment. Downstream IVCP did not change (Fig. 1d). Urine volumes and GFR in both kidneys were immediately decreased after ligation, and gradually recovered and returned to baseline in the control kidneys. However, urine volumes and GFRs remained decreased throughout the experiment in the congestive kidneys (Fig. 1e,f).

To evaluate the recovery of blood flow after the release of ligation, a vascular clip was used. Optical fibers were inserted into the both renal cortical or outer medullary regions for measurement of cortical blood flow (CBF) and medullary blood flow (MBF). For RIHP measurement, catheters were placed in the cortex (Fig. 1g). The IVC was clamped between the renal veins with vascular clips under anesthesia. Both CBF and MBF in the congestive kidneys decreased after clamping, but returned to baseline immediately after removal of the clip. However, CBF and MBF in the control kidneys tended to decline slightly, but the extent of reduction was not as large as the reduction in left RBF (Fig. 1h,i). Although there was a tendency towards a reduction of RIHP in both kidneys soon after ligation because of decreases in blood pressure, sustained increases in RIHP were only observed in the congestive kidneys (Fig. 1j). To investigate whether renal congestion leads to hypoxia as the blood flow decreases, immunochemical detections using pimonidazole, a hypoxia detector, were performed. Pimonidazole deposits were observed mainly in medullary thick ascending limbs (mTAL) near the descending vasa recta (DVR) in the congestive kidneys only (Fig. 1k).

### Subacute animal experiments

#### Biochemical analysis

**Figure 2:**
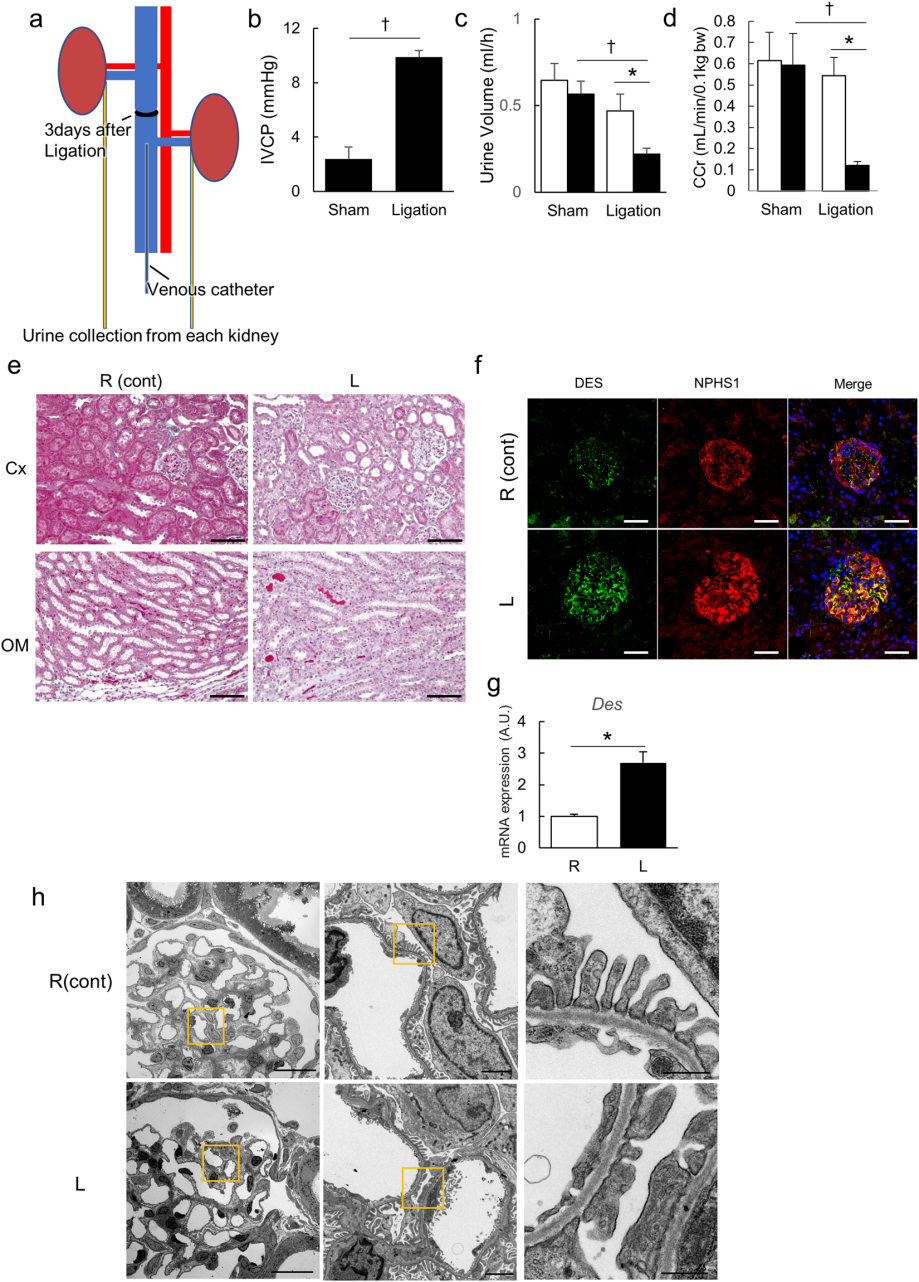
Biochemical analysis and histology in the subacute phase. (**a**) The schema of the biochemical analysis in the subacute phase. The catheters were detained for IVCP measurement and urine collection from each kidney. (**b**) Upstream IVCP of IVC ligated rats was compared with sham-operated rats. ^†^p < 0.05 versus sham-operated group; *t*-test. (**c**) Urine volume and (**d**) CCr are shown. Sham n = 4; Ligation n = 6. Data are presented as the means + SEM. *p < 0.05 versus control kidneys, ^†^p < 0.05 versus sham-operated group; Tukey’s test. In (**c,d**), ▫ indicates the control kidneys and ▪ indicates the congestive kidneys. (**e**) Elastica Masson staining of kidneys in the subacute phase. Scale bar, 100 µm. (**f**) Immunofluorescent analysis of DES and NPHS1. Scale bar, 25 µm. (**g**) mRNA expression level of *Des*. The result is compared between the congestive kidneys and control kidneys. n = 5–7. Data are presented as the means + SEM. *p < 0.05 versus control kidneys, ^†^p < 0.05 versus Sham group; Tukey’s test. (**h**) Observation by transmission electron microscope. Scale bars are 20 µm, 2 µm, and 500 nm from the left. IVCP: inferior vena caval pressure; Cx: cortex; OM: outer medulla; R (cont): control right kidney; L: congestive left kidney; DES: Desmin. NPHS1: Nephrin.

No rats died during the experimental period. We reviewed the histology and molecular analysis on postoperative day 3 because collateral circulation was considerably increased on day 7. Three days after surgery, we measured the IVCP in the subacute phase and obtained plasma and urine under anesthesia. The catheters were detained for IVCP measurement and urine collection from each kidney (Fig. 2a). Upstream IVCP in the ligation group was still significantly higher than in the sham-operated group (Fig. 2b). Urine volumes from the congestive kidneys were still lower in the ligation group after 3 days compared to the sham-operated group (Fig. 2c). Creatinine clearance (CCr) of the congestive kidneys was still lower in the ligation group after 3 days compared to the sham-operated group (Fig. 2d). Serum creatinine (Cr) and urea nitrogen (UN) in the ligation group were higher than in the sham-operated group (Supplemental Table S1). CCr, UN, and inorganic phosphorus were lower in the congestive kidneys compared to the right control kidneys (Supplemental Table S1). Furthermore, increased urinary albumin (Alb) excretion was observed in the Alb/Cr ratio (Supplemental Table S1).

#### Morphological analysis

Extension of the tubules and extracellular space expansions were visualized using Elastica Masson staining (Fig. 2e). Staining intensity and mRNA expression levels of desmin (*Des*) were higher in the congestive kidneys compared to the control kidneys (Fig. 2f,g). The glomerular structure, including the podocytes, was maintained in the control kidneys. However, foot process effacement and disruption of the slit diaphragm were observed in the congestive kidneys (Fig. 2h).

#### Microarray analysis

**Figure 3:**
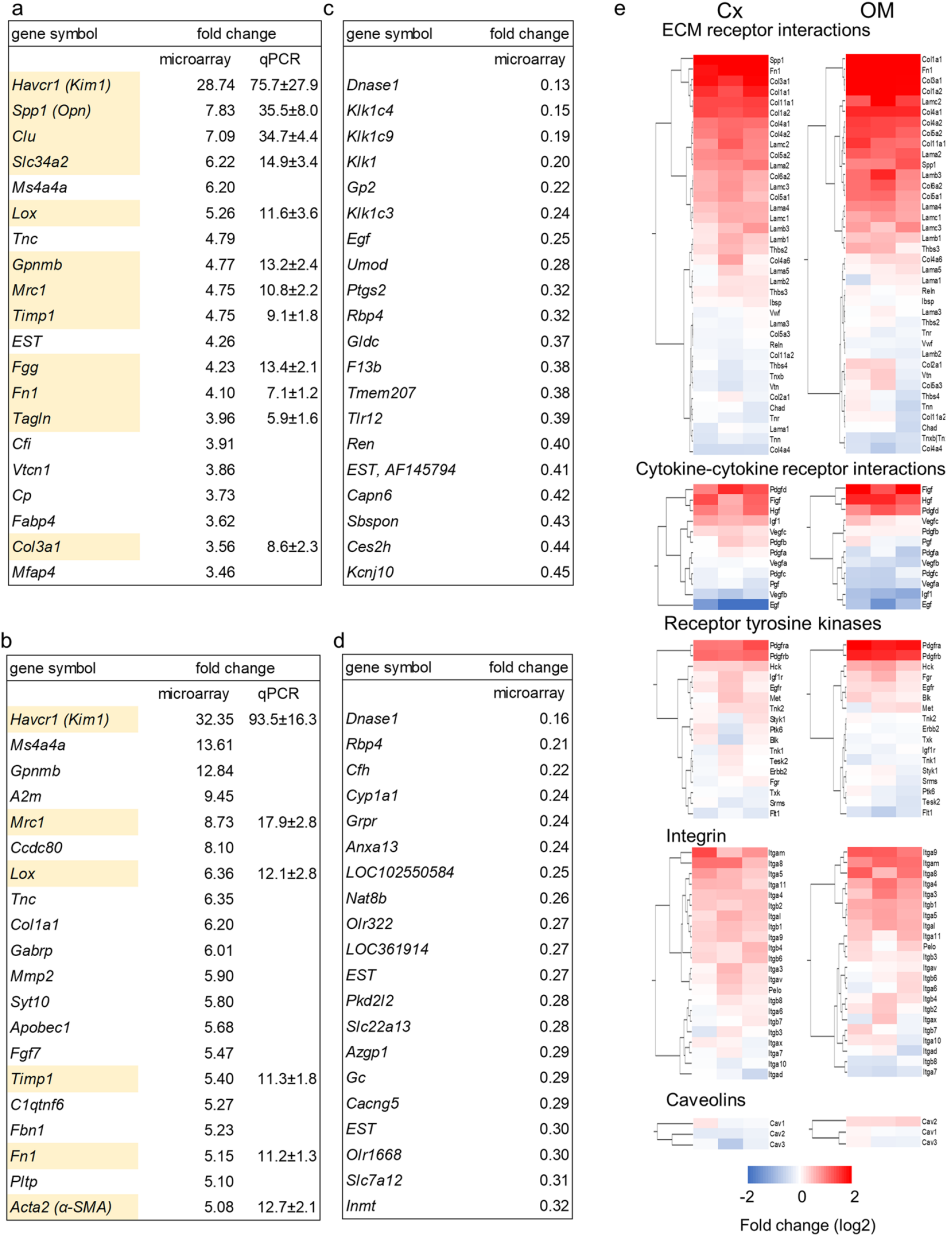
Microarray analysis. (**a–d**) Upregulated genes in the congestive kidneys compared to the control kidneys in the cortex (**a**) and medulla (**b**). Downregulated genes in the congestive kidneys compared to the control kidneys in the cortex (**c**) and medulla (**d**). The values were expressed as the fold change of the left expression divided by the right, n = 3. EST: expression sequence tag and subsequent symbols in GenBank ID. Aliases are presented in parentheses. Genes in yellow background cells are those for which the expression patterns were confirmed by qPCR. Expression of mRNA was corrected by *Gapdh* mRNA level in the cortex and medulla. n = 7. Data are presented as the means + SEM. (**e**) Receptors and ligands of the focal adhesion pathway were extracted from microarray results. Congestive kidney expression level relative to the control kidney in logarithm is shown. Each line represents each gene. Each column represents each individual (n = 3).

We performed microarray analyses of the total RNA extracted from the renal cortex and medulla 3 days after ligation. Gene ontology analyses revealed increases in extracellular factors and decreases in vasoconstriction regulation (Supplemental Tables S2–5). Notably, markers of extracellular matrix expansion and tubular injury were elevated in the congestive kidneys compared to control kidneys (Fig. 3a,b). Real-time quantitative PCR (qPCR) was performed to confirm the microarray analysis with respect to extracellular factors and kidney injury markers. The expression patterns were identical in all RNAs analyzed by qPCR, and the expression levels were significantly higher in the congestive kidneys than in the control kidneys (Fig. 3a,b). In contrast, genes such as DNAase, kallikreins, renin and uromodulin (Umod) were downregulated (Fig. 3c,d).

The number of genes with increased expression was the largest in the focal adhesion pathway in a microarray pathway analysis using TAC 4.0 software (Table 1). The focal adhesion pathway includes receptors, ligands, and signal genes (Fig. 3e, Supplemental Fig. S1). When we focused on the pathway’s receptor and ligand genes, we observed that platelet derived growth factor receptors (PDGFRs) and ligands were highly expressed in the congestive kidneys (Fig. 3e), which indicates that these molecules may play a role in the pathogenesis of renal congestion.

**Table 1:** Top 20 pathways derived by TAC4.0.

Pathway	Total	Up	Down	p-value	Significance*
Focal Adhesion	83	68	15	<0.001	10.1
MAPK Signaling Pathway	73	60	13	0.004	2.4
EGFR1 Signaling Pathway	63	53	10	<0.001	4.3
Electron Transport Chain	59	1	58	<0.001	15.4
Myometrial Relaxation and Contraction Pathways	53	47	6	<0.001	3.7
Regulation of Actin Cytoskeleton	52	44	8	<0.001	3.5
Nuclear factor, erythroid-derived 2, like 2 signaling pathway	52	28	24	0.182	0.7
TNF-alpha NF-kB Signaling Pathway	51	41	10	0.036	1.4
TGF-beta Receptor Signaling Pathway	51	41	10	<0.001	3.2
B Cell Receptor Signaling Pathway	51	47	4	0.003	2.6
Insulin Signaling	50	37	13	0.007	2.2
T Cell Receptor Signaling Pathway	50	43	7	<0.001	4.6
Calcium Regulation in the Cardiac Cell	48	36	12	0.002	2.8
Androgen Receptor Signaling Pathway	45	34	11	<0.001	5.1
Wnt Signaling Pathway NetPath	39	33	6	<0.001	3.0
IL-3 Signaling Pathway	38	35	3	<0.001	3.3
Cytoplasmic Ribosomal Proteins	36	35	1	<0.001	4.0
Oxidative phosphorylation	36	0	36	<0.001	9.7
Integrin-mediated cell adhesion	35	30	5	0.003	2.5
Adipogenesis	35	23	12	0.209	0.7

*Significance means −10 logarithm of p-value.

#### The ameliorating effect of decapsulation

**Figure 4:**
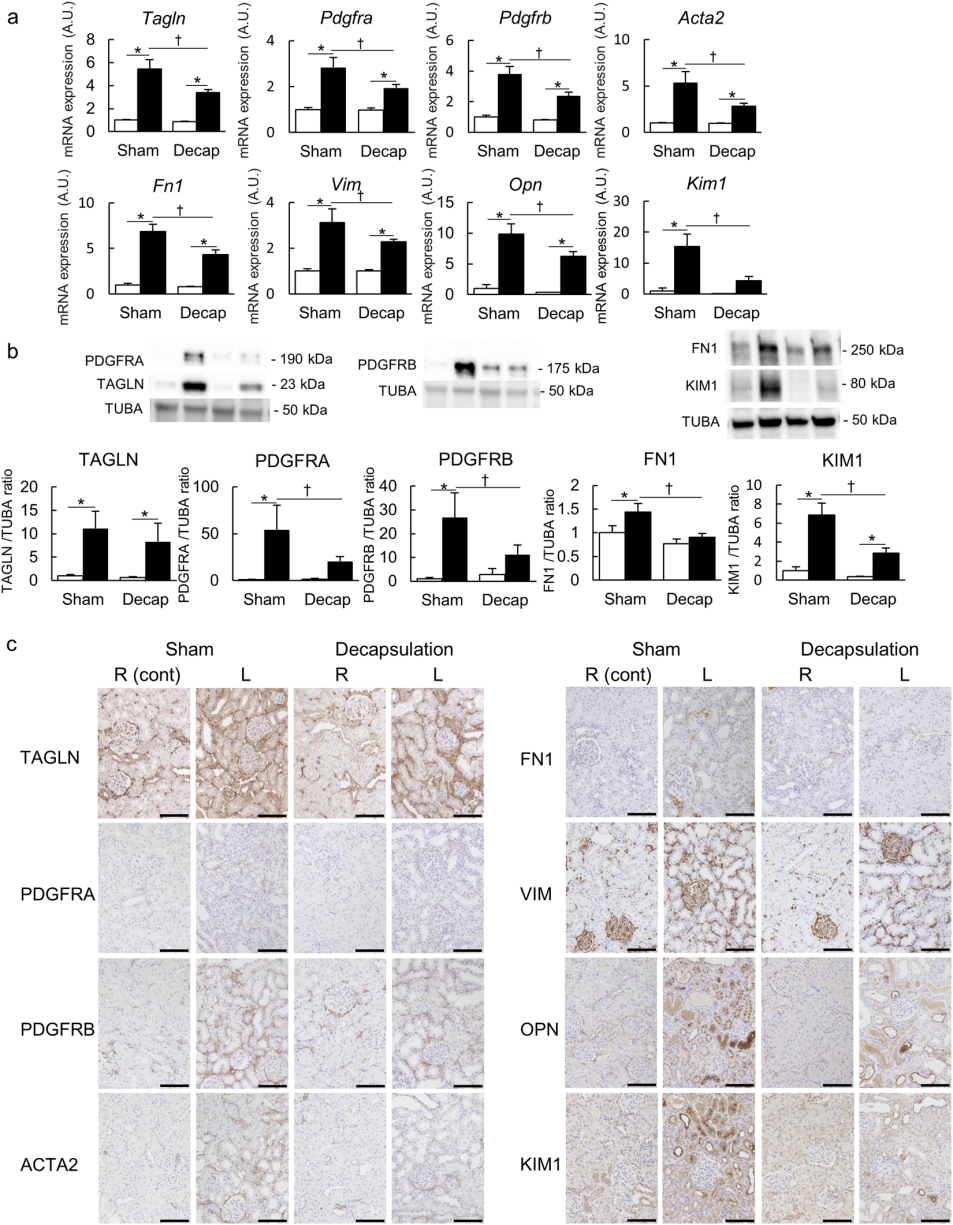
Molecular analysis of the cortex. (**a**) mRNA expression level of *Tagln, Pdgfra, Pdgfrb, Acta2 (α-Sma), Fn1, Vim, Opn* and *Kim1* in the cortex. (**b**) Western blotting analysis of TAGLN, PDGFRA, PDGFRB, FN1, and KIM1. In these analyses, the left renal decapsulated IVC ligation model (Decap) was compared with the sham IVC ligation model (Sham). Sham n = 5, Decap n = 7. Data are presented as the means + SEM. *p < 0.05 versus control kidneys, ^†^p < 0.05 versus Sham group; Tukey’s test. ▫ indicates the control kidneys and ▪ indicates the congestive kidneys. Full-length blots are presented in Supplementary Fig. S8. (**c**) Immunostaining of TAGLN, PDGFRA, PDGFRB, α-SMA, FN1, VIM, OPN and KIM1. Scale bar, 100 µm. R (cont): control right kidney; L: congestive left kidney.

**Figure 5:**
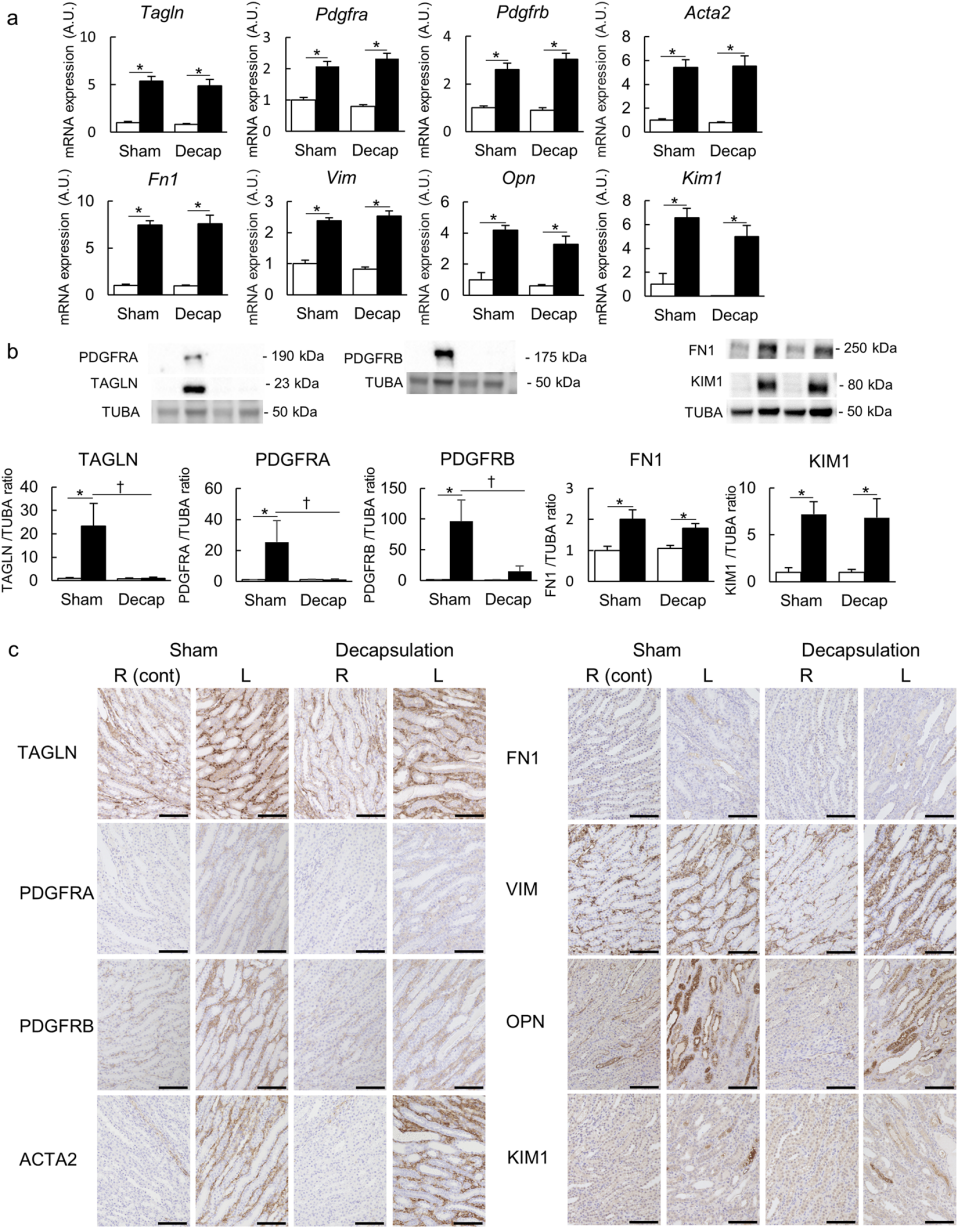
Molecular analysis of the medulla. (**a**) mRNA expression level, (**b**) western blotting analysis, and (**c**) immunostaining in the medulla of the same molecules analyzed in the cortex. The left renal decapsulated IVC ligation model (Decap) was compared with the sham IVC ligation model (Sham). Sham n = 5, Decap n = 7. Data are presented as the means + SEM. *p < 0.05 versus the control kidneys, ^†^p < 0.05 versus the Sham group; Tukey’s test. ▫ indicates the control kidneys and ▪ indicates the congestive kidneys. Scale bar, 100 µm. R (cont): control right kidney; L: congestive left kidney. Full-length blots are presented in Supplementary Fig. S8.

To determine whether RIHP was responsible for the pathogenesis of renal congestion, we performed qPCR on a left renal decapsulated IVC-ligation model (Decap) and compared it to a sham-operated IVC-ligation model (Sham). Most of the markers of profibrosis and tubular injury were elevated in the congestive kidneys compared to the control kidneys in the Sham and Decap groups. However, the levels of renal congestion-induced profibrosis and kidney injury markers were significantly attenuated by decapsulation in the cortical region only (Fig. 4a, Supplemental Fig. S2a). The benefit of decapsulation was not detected with most genes we investigated in the medullary region (Fig. 5a, Supplemental Fig. S2b).

Upregulation of transgelin (TAGLN), PDGFRA and PDGFRB were confirmed by western blotting, and all were ameliorated by decapsulation (Figs 4b, 5b). Significantly increased expressions of fibronectin1 (FN1) and kidney injury molecule-1 (KIM1) in the congestive kidneys compared to control kidneys were also detected by western blotting. These proteins were significantly decreased by decapsulation, but only in the cortex (Figs 4b, 5b).

Immunohistochemical analysis revealed hyperplasia of the extracellular matrix and renal tubular injury in the congestive kidneys (Figs 4c, 5c). The pericyte-myofibroblast transition markers, TAGLN, PDGFRA and PDGFRB were stained in the interstitial space of the congestive kidneys; however, these proteins were decreased by decapsulation. The extracellular matrix markers, actin-alpha2-smooth muscle (ACTA2, α-SMA), FN1 and vimentin (VIM) positive areas were increased in the interstitial space of the congestive kidneys. The tubular injury markers, osteopontin (OPN) and KIM1 were strongly stained in the atrophic tubules of the congestive kidneys. The decapsulation amelioration effects for extracellular matrix injury and renal tubular injury were only observed in the cortical regions, similarly to the results of the qPCR.

#### Pericyte detachment

**Figure 6:**
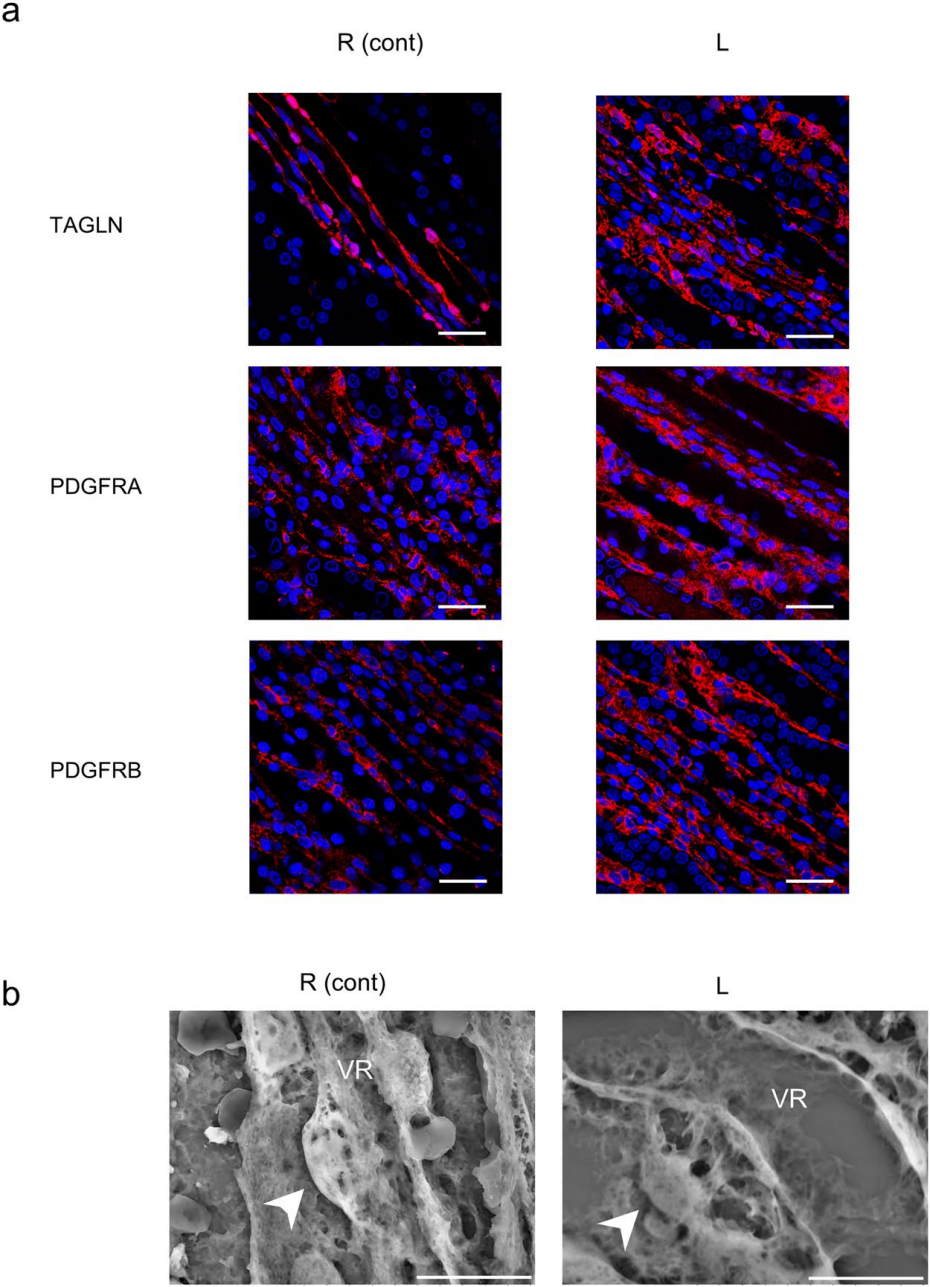
Pericyte detachment. (**a**) Immunostaining of TAGLN, PDGFRA, and PDGFRB in the descending vasa recta. Scale bar, 25 µm. (**b**) Structure of pericytes (arrow head) in the descending vasa recta (VR) by scanning electron microscopy. Scale bar, 10 µm. R (cont): control right kidney; L: congestive left kidney.

Since TAGLN and PDGFRs are responsible for pericyte-myofibroblast transition, we investigated whether TAGLN and PDGFRs were expressed in the pericytes of the vasa recta and surrounding interstitial cells. TAGLN and PDGFRs were highly expressed in the pericytes and surrounding interstitial cells of the congestive kidneys compared to the control kidneys (Fig. 6a). Calponin (CNN1), a myofibroblast differentiation marker, was expressed around the vasa recta only in the congestive kidney (Supplemental Fig. S3). LVSEM revealed pericyte detachment with expansion of the vasa recta in the congestive kidneys, while there were no detached pericytes in the control kidneys (Fig. 6b).

## Discussion

We report here a novel rat renal congestion model based on IVC ligation between the renal veins. Only a few reports have investigated the physiological role of renal congestion by IVC ligation above the renal veins^12,19^. To our knowledge, this is the first report of ligation between the renal veins, and we have determined the underlying mechanisms of renal congestion using physiological, histological, and molecular analyses. Increases in IVCP and decreases in GFR, UV, and blood flow were detected in the acute phase after ligation. In the subacute phase, IVCP of the ligation group was still higher than in the sham group, although acute phase recovery did occur. CCr and UV of the congestive kidneys were still lower than the control kidneys in the subacute phase. Results of the molecular and histological analysis suggested a role for pericyte-mesenchymal transition in the pathogenesis of renal congestion. We successfully demonstrated a novel mechanism, whereby pericyte loss triggered renal interstitial injury.

### A novel rodent model for the renal congestion

Recently, increased IVCP has received attention as a major factor in renal dysfunction^3^, but the mechanism underlying this effect is not yet known. IVC ligation is the simplest model for increasing IVCP. However, IVC ligation above the renal veins is severe and sometimes lethal^20^. Decreases in renal function cause potentially lethal hyperkalemia and hyponatremia^12^. In our model, right kidney GFR recovered within 1 h, and no changes in CCr, or histological changes by postoperative day 3 were observed; thus, such problems would not occur. In addition, no rats died during the experiments. Furthermore, the advantage of this model is that it allows us to compare the congestive kidneys to the control kidneys. With this comparison, we can compare molecules and renal mechanisms without confounding effects related to different genetic backgrounds and circulating factors, including systematic blood pressure and vasoactive substances.

### RBF and RIHP in renal congestion

Reduced CBF and MBF were monitored in the congestive kidneys after IVC ligation. RVP rose with increased IVCP. It has been previously described that the increase in IVCP causes the reduction of RBF^8,21^ and our results support these reports. In dogs, RBF is controlled in the normal range and renal function remains stable until an RVP of 18 to 20 mmHg is reached^22^. We could not directly measure RVP in our rat model; thus, IVCP was measured instead at the level of each renal vein. Our results, taken together with previous reports, indicate that RBF decreases due to an increase in RVP as the IVCP rises. This is also consistent with our finding that reduced CBF and MBF occur together with increased RIHP. However, CBF and MBF immediately decreased after ligation, whereas RIHP gradually increased. Thus, there might be another mechanism involved in decreasing local blood flow. Since renal capillary pressure may increase as a result of elevated RVP, local blood flow could be reduced by increases in downstream pressure. Meanwhile, capillary pressure elevations could cause increases in GFR. However, our results showed the opposite; the renal tubules were compressed as RIHP rose^5^, causing GFR reduction.

### Molecules involved in the pathogenesis of renal congestion

The highly upregulated genes in renal congestion, detected by microarray analyses, were mainly extracellular matrix expansion and tubular injury markers. These increases may not be the cause, but rather the result of the injury. Even so, these could be used as markers of renal congestion, since protein expressions of these genes were also elevated. KIM1 showed the highest expression in our model, and is thought to be the strongest predictor of worsening renal failure in patients with CHF^23^. Other highly expressed molecules are also candidate markers of renal congestion although they may not be specific enough. Further investigation is necessary to determine the most effective molecular markers of renal congestion. Especially, pericyte damage markers should be noteworthy.

Pathway analysis revealed that focal adhesion pathways, which consist of extracellular matrix receptor interactions, receptor tyrosine kinases, and integrins, are primarily upregulated in congestive kidneys, as shown in Fig. 3e. Focal adhesion pathways cause cell proliferation, which is considered to be a major factor in renal fibrosis^24^. The PDGFR is one of the receptors in this pathway and has been reported to induce pericyte-myofibroblast transition in the kidney^25^. Together with the microscopic findings, we can suggest that pericyte detachment induced by physical stimulation activates PDGFR, which was the main factor in extracellular matrix expansion in this study.

### The ameliorating effect of decapsulation

Two beneficial effects of decapsulation have been previously described. One is the suppression of RIHP^26,27^, and the other is the improvement of renal tissue hypoxia^28^. Our results indicated that tubulointerstitial injury was dependent on RIHP. This is consistent with previous reports describing mechanical stimulation-induced fibrosis^29^. Although there is no evidence that decapsulation alters total blood flow^30^, hypoxia could be improved by amelioration of the microcirculation. The level of tubulointerstitial injury and most of the profibrotic markers were ameliorated only in the cortex, while contrastingly, pericyte-myofibroblast transition-related proteins, such as PDGFRs and TAGLN, were ameliorated mainly in the medulla. The different results between the cortex and medulla might be due to differences in the RIHP response to the decapsulation. One report has suggested that RIHP of the medulla was not reduced by decapsulation, despite reductions in the cortex^26^.

### Tubular injury in renal congestion

Several factors could be involved in the mechanism of tubular injury in our model. Firstly, reduced RBF may decrease GFR and urine flow. Urine flow induces primary cilium-dependent autophagy, which is required for homeostasis of kidney epithelial cells^31^. Urine flow in our model did not recover even after 3 days. Reduced RBF may also cause tubular hypoxia, especially in the proximal tubules and mTAL which require the most oxygen. Secondly, decreased expression of *Umod* could cause tubular injury. Mutations in *Umod* are reported to cause interstitial disease^32^, although the function of this molecule has not been fully clarified. In an experiment using an *Umod*-deficient mouse model, *Umod* was found to be involved in epithelial transport regulation^33^. Indeed, decreases in *Umod* expression were observed in the microarray analysis we conducted (Fig. 3c). Thirdly, increases in RIHP could induce tubular compression^34^ and tubular injury. Expansion of the AQP2-positive collecting duct was observed in the congestive kidneys (Supplemental Fig. S4). Although the precise mechanisms have not been sufficiently described in the literature, intercalated cells of collecting ducts were detached after ischemia^35^, which could cause luminal expansion. Decapsulation could release the tubular compression, and decrease tubular injury.

### Podocyte injury in renal congestion

Podocyte injury and slit diaphragm disruption were observed in our model, resulting in albuminuria. Albuminuria has been reported in patients with CHF^36^. Rafiq *et al*. described that intrarenal angiotensin II is overexpressed in an aortic regurgitation-induced cardiac volume overload model, contributing to podocyte injury and albuminuria^37^. However, the renin-angiotensin-aldosterone system was not necessarily activated in our model (Supplemental Fig. S5). Other injuries and stresses, such as exposure to toxins or increases in intracapillary pressures, could be the cause of podocyte injury^38,39^.

### Mechanism of interstitial injury in renal congestion

We found luminal DVR expansion, but not much in peritubular capillaries. Additionally, pericyte loss was observed in the congestive kidneys together with extracellular matrix expansion. Based on this observation, we initially assumed that pericyte detachment triggered fibrogenesis. Detached pericytes are transdifferentiated into myofibroblasts and proliferate, causing fibrosis^17^. Pericytes surround the DVR and constrict it to maintain medullary hemodynamics^40^. Thus, regulation of medullary circulation could be altered by pericyte detachment, resulting in hypoxia in the surrounding tissue. Pericytes are reported to detach under stress^41^, and although the underlying mechanisms are unclear, hypoxia, reactive oxygen species, RIHP, and capillary expansion pressure are thought to act as compound triggers of pericyte detachment. We do not have enough data showing which possibility is the highest, however we hypothesized that hypoxia might be the key factor underlying damage. Pimonidazole was stained in the mTAL of congestive kidneys but not in the pericytes. Mori *et al*. previously demonstrated that superoxide is diffused from mTAL to pericytes^42^. Thus, one possible mechanism is that hypoxia of mTAL triggers superoxide production, which induces pericyte detachment.

The PDGF pathway is a representative pathway of pericyte-myofibroblast transition^17,43^. Activation of the PDGFRs is detected in ureteral obstruction or ischemia reperfusion mouse models, and causes fibrosis^25^. In this study, we observed increased expression of PDGFRs around the DVR, indicating the presence of pericyte-myofibroblast transition.

Although expansion of the DVR was observed in the present model, the DVR diameter does not expand when one increases renal perfusion pressure, which is another model that raises RIHP^44^. On the other hand, pericyte loss induces DVR expansion^45^. The literature suggests that pericyte detachment precedes expansion of the DVR in this model, but we cannot deny the possibility that expansion of the DVR occurs first.

Thus, with consideration to previous results, one novel finding of the current study is that pericytes play a key role in the pathogenesis of renal congestion.

### Limitations

Because of collateral circulation in this model, kidney injury started to recover within just 1 week (Supplemental Fig. S6). There are many reports of collateral generation induced by venous ligation^46,47^. IVCP elevation to around 20 mmHg decreased to around 10 mmHg after only 3 days. Thus, this model might not be suitable for long-term observation. Because of this short period, chronic fibrosis might not develop despite of highly expression of acute profibrotic markers. Laser doppler measurement of local blood flow is useful for change over time, but we cannot use it for direct comparison of absolute values. The little difference of the optical fiber position can cause big difference of the value. Therefore, we cannot compare renal blood flow between right and left directly. Moreover, almost all (58/59) genes in the electron transport chain pathway in the mitochondria were downregulated according to the microarray analysis, and mitophagosomes were observed in the congestive kidneys (Supplemental Fig. S7). These results imply that mitochondrial dysfunction was related to the pathogenesis of this model and further studies are required to elucidate its role. Additionally, because the heart and right kidney function in this model were almost normal, neuroendocrine system functions might be different from actual CHF. However, this is also an advantage of our model, because it enables the direct evaluation of renal congestion, independent of neurohormonal factors.

## Conclusion

**Figure 7:**
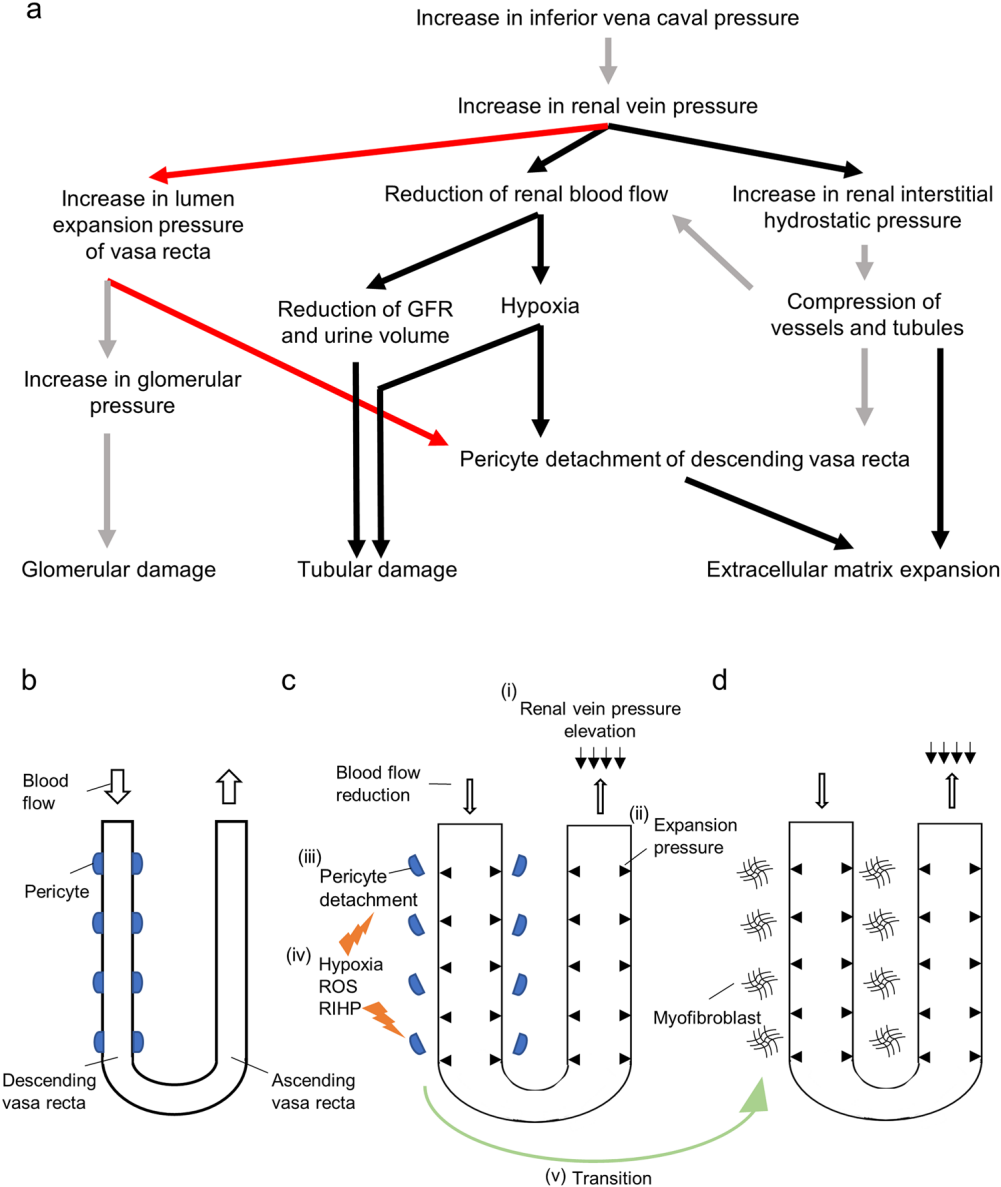
Proposed mechanism of renal injury on renal congestion. (**a**) Schema of the proposed mechanism of renal failure in this model. Pericyte detachment is caused by multiple factors, such as mechanical pressure, hypoxia, and reactive oxygen species. Pericyte detachment might be a trigger of pericyte myofibroblast transition and induce extracellular matrix expansion. Red arrow: demonstrated in the present study for the first time; black arrow: reproduced previous research; gray arrow: already demonstrated mechanisms. (**b**–**d**) show the schema of pericyte detachment and pericyte myofibroblast detachment transition. (**b**) Base state, (**c**) Renal vein pressure elevation induces blood flow reduction (i) and expansion pressure elevation (ii), which induces pericyte detachment (iii). Hypoxia, reactive oxygen species, and mechanical pressure (iv) induce pericyte myofibroblast transition (v).

As summarized in Fig. 7, an increase in IVCP induces a reduction of RBF together with RIHP. With compression of peritubular capillaries and tubules, hypoxia and physical stress induce pericyte detachment and podocyte injury, which could result in the extracellular matrix expansion and tubular injury.

## Methods

### Animals

All animal experiments were in accordance with the National Institutes of Health Guide for the Care and Use of Laboratory Animals, and were approved by the Tohoku University Animal Experiment Committee (registration number: 2017MdA-318). Male Sprague-Dawley rats (7- to 9-weeks old, 250–350 g, Japan SLC, Shizuoka, Japan) were housed in environmentally controlled rooms under a 12-h light/dark cycle. Rats had free access to a pelleted diet (Labo MR Stock, Nosan corporation, Yokohama, Japan) and water. Animal experiments were performed after at least 3 days of acclimation. Sample size was chosen based on previously published experiments performed under similar conditions^48–50^.

### Subacute phase IVC ligation for the biochemical and molecular analyses

All rats were anesthetized with ketamine (30 mg/kg body weight (bw), intramuscularly) and xylazin (2.0 mg/kg bw, intramuscularly) before surgery. After midline abdominal incision, the IVC between the two renal veins was carefully exposed using a cotton swab, and the intestines were covered with standard cotton gauze soaked in warm saline. The IVC between the renal veins was ligated and the abdominal wall and skin were closed using silk suture 3–0. No anatomical vein abnormalities were observed. After surgery, penicillin (300,000 U/kg bw, intramuscularly) and buprenorphine (0.05 mg/kg bw, subcutaneously) were administered, and the rats were monitored on a warm table until they recovered from the anesthesia. Sham-operated rats were treated similarly, but without ligation.

Three days after surgery, we measured the IVCP in the subacute phase and obtained plasma and urine under anesthesia for biochemical analyses (sham-operated group, n = 4; ligation group, n = 6). Rats were anesthetized with ketamine (20 mg/kg, intramuscularly) and inactin (50 mg/kg, intraperitoneally), and placed on temperature-controlled (37 °C) surgical tables. PE 240 catheters were inserted into the tracheas to maintain respiration. PE 50 catheters were inserted into the left femoral veins to measure IVCP, left cervical veins for infusion, and bilateral ureters to collect urine from each kidney. Venous blood was collected from the cervical vein catheters and centrifuged (16,060 g, 4 °C, 10 min). Saline containing 2.0% bovine serum albumin (BSA) was infused through the cervical vein catheter at 1.2 mL/100 g bw/h. The flow rate here was different from the flow rates in other acute experiments performed in this study so that we could obtain enough urine for biochemical examinations. The urine samples were collected separately from each kidney for 2 h. IVCP was measured for 30 min using the PowerLab system (AD Instruments, Sydney, Australia). Biochemical examinations of plasma and urine were performed at Nagahama Life Science Laboratory (Nagahama, Japan).

For molecular analysis, the renal tissues were removed under anesthesia and the rats were euthanized three days after surgery. Both kidneys were immediately sectioned and fixed with 10% neutral-buffered formalin for histological analyses, and then divided into the cortex and medulla for RNA and protein analyses. The RNA samples were stored in RNA Later (Invitrogen, Carlsbad, CA) and stored at −30 °C. The protein samples were snap frozen in liquid nitrogen and stored at−80 °C.

To attenuate RIHP elevations^44^, left renal capsulotomies were performed (since the right renal capsule was intact) 5 days before IVC ligation (Sham, n = 5; Decap, n = 7). Under anesthesia, incisions were made in the left abdomens to expose the left kidneys. Next, the left renal capsules in the decapsulation group were totally removed. The kidneys were then returned to the retroperitoneal space and the abdominal walls and skin were closed. Sham-operated rats were treated similarly, but without decapsulation.

### Acute phase IVC ligation for the physiological analysis of renal hemodynamics

#### Measurement of arterial blood pressure, inferior vena cava pressure, and glomerular filtration rate

Anesthesia and respirations were performed as above. PE 50 catheters were inserted into the left femoral artery to measure ABP, the bilateral femoral veins to measure IVCP, the left cervical vein for infusion, and bilateral ureters to collect urine from each kidney. Saline containing 2.0% BSA was infused at a rate of 1.0 mL/100 g bw/h. Upstream and downstream catheters to measure IVCP were maintained at the junctions of both renal veins and the vena cava. ABP and IVCP were measured using the PowerLab system. GFR was measured using inulin-fluoresceinyl isothiocyanate clearance as previously described^51^. To measure GFR, saline containing 2.0% BSA and 2.0 mg/mL inulin-fluoresceinyl isothiocyanate was infused at a rate of 1.0 mL/100 g bw/h. After the animals had rested for at least 1 h for equilibration, we measured ABP, PR, and IVCPs for 1 h and recorded these as baseline values. Next, the IVC between the renal veins was totally ligated with silk suture including the downstream IVCP catheter. ABP, PR, and IVCPs were continuously measured for 2 h, and 500 μL of blood was collected from the left femoral artery catheter before and after each experiment. Urine from each kidney (n = 5) was collected at 30-min intervals.

#### Measurement of renal cortical blood flow, medullary blood flow, and renal interstitial hydrostatic pressure

Anesthesia and respirations were performed as above. PE 50 catheters were inserted into the left femoral artery to measure ABP, the left cervical and right femoral veins for infusion, and the bladder to collect urine. Kidneys were isolated and placed in a plastic kidney cup to fix their positions. Optical fibers were inserted into the cortical or outer medullary regions of both kidneys, and renal CBF (n = 4) and MBF (n = 5) were measured using laser tissue (Doppler) flowmeters (OMEGAFLO, Omega Wave, Tokyo, Japan). Saline containing 2.0% BSA was infused from the cervical vein catheter at a rate of 1.0 mL/100 g bw/h. Saline was infused from the right femoral vein at a rate of 0.5 mL/h. After the animals had rested for at least 1 h for equilibration, we measured CBF and MBF for 1 h and recorded these as baseline values. Next, the IVC between the renal veins was clamped for 50 min using a micro-vascular clip (Fine Science Tools 18055–05, North Vancouver, Canada), and the clip was then removed. The CBF and MBF were continuously measured while clamped, and for 30 min after clamp removal. At the end of experiment, Nω-Nitro-L-arginine methyl ester hydrochloride (L-NAME) (6.0 mg/kg bw/h) was infused for 30 min intravenously as a positive control; L-NAME does not affect CBF but decreases MBF^40,48^. The data without L-NAME response were excluded from the analysis. After the experiment, the kidneys were hemisected, and the position of the optical fiber tip was confirmed. RIHP (n = 4) was measured as previously described^26^. The PE 50 was plugged using polyethylene matrix materials and filled with 10% heparinized saline. The catheters were implanted into the cortex. RIHP was measured using the PowerLab system.

### Renal hypoxia analysis

To evaluate hypoxic effects of renal congestion, we conducted immunohistochemical analyses using the Hypoxyprobe^TM^-1 kit (HP1-100, Hypoxyprobe, Inc., Burlington, MA) according to the manufacturer’s instructions. Under anesthesia, pimonidazole HCl (Hypoxyprobe^TM^-1, 60 mg/kg bw, n = 3) was administered intraperitoneally immediately following. IVC ligation between the renal veins. Two hours later, both kidneys were removed and fixed with 10% neutral-buffered formalin (Wako Pure Chemical Industries Ltd., Osaka, Japan).

### Microarray analysis

Total RNA from the renal cortex and medulla were extracted using ISOGEN (Nippon Gene, Tokyo, Japan). After purification by column chromatography, microarray assays and statistical analyses were performed using GeneChip^TM^ Rat Gene 2.0 ST Array (Affymetrix, CA) at Riken Genesis (Kanagawa, Japan) (n = 3). Pathway analyses were processed with Transcriptome Analysis Console version 4.0 software (TAC 4.0; Thermo Fisher Scientific, Waltham, MA).

### Quantification of RNA expression levels

Two µg of total RNA was used as a template for cDNA synthesis using SuperScript III First-Strand Synthesis SuperMix (Invitrogen). An aliquot of synthesized cDNA was used as a template for qPCR, using the CFX96 Touch Real-Time PCR Detection System (Bio-Rad, Hercules, CA). The target cDNAs were amplified with specific primers (Supplemental Table S6, purchased form Takara Bio, Otsu, Japan) using SYBR Premix Ex Taq reagents (Takara Bio). The relative mRNA expression levels were normalized by glyceraldehyde-3-phosphate dehydrogenase (GAPDH).

### Histological analysis

Formalin-fixed paraffin-embedded renal tissues were sliced into 2.0 μm-thick sections, then deparaffinized with xylene and hydrated with 100% ethanol. The antigens were retrieved by microwave heating for 5 min in 10 mmol/L citrate buffer for PDGFRs, TAGLN, VIM, KIM1, CNN1 and Aquaporin-2 (AQP2) staining or 5 mmol/L ethylenediaminetetraacetic acid buffer for DES and Nephrin (NPHS1). The antigens were reacted with antigen-specific antibodies (Supplemental Table S7) overnight at 4 °C. Sections were reacted with Histofine Simple Stain MAX PO (Nichirei, Tokyo, Japan) or fluorescent labeled secondary antibodies (Alexa Fluor 488 and Alexa Fluor 555, 1:500, Molecular Porbes, Eugene, OR) for 30 min at 37 °C. Immunostaining was developed using 3,3′-diaminobenzidine (Dojindo, Kumamoto, Japan), counterstained with hematoxylin, and photographed by a light microscope (BZ-9000, KEYENCE, Tokyo, Japan). Immunofluorescent sections were counterstained with Hoechst 33342 (Molecular probes) and observed by a TCS-SP8 confocal microscope (Leica Microsystems, Wetzlar, Germany).

### Transmission electron microscopy

In collecting samples for electron microscopy, perfusion-fixation of the kidneys with 4% paraformaldehyde was performed before nephrectomy. Renal tissues from IVC-ligated rats were cut into 2.0-mm^3^ pieces. Electron microscopy was then performed as previously reported^52^. The samples were fixed in 2.5% glutaraldehyde and 2.0% paraformaldehyde in 0.2 mol/L cacodylate buffer (pH 7.4), followed by post-fixation in 1.0% osmium tetroxide in 0.08 mol/L cacodylate buffer and 6.0% sucrose. After dehydrating with ethanol and propylene oxide, the tissues were mounted on epoxy resin (EPON812, DDSA, MNA, DMP-30; TAAB Laboratories, Berks, UK). The sections were cut into 90 nm-thick sections using a Leica EM UC6 (Leica Microsystems) and visualized with a JEM-1400 electron microscope (JEOL, Tokyo, Japan).

### Low-vacuum scanning electron microscopy

Low-vacuum scanning electron microscopy (LVSEM; Miniscope TM3030, Hitachi High-Technologies, Tokyo, Japan) was used to examine the structure of the outer medullary vasa recta. Four-μm sections were deparaffinized, dehydrated, and stained with a Pt-blue solution (TI-blue small kit, Nisshin EM, Tokyo, Japan) for 15 min as previously described^53^. After washing with distilled water, the sections were observed under LVSEM at an acceleration voltage of 15 kV and 30 Pa.

### Western blotting

The kidney tissues were homogenized in radioimmunoprecipitation assay buffer (Cell Signaling Technology, Danvers, MA) containing 1.0 mmol/L phenylmethylsulphonyl fluoride (Thermo Fisher Scientific) and protease inhibitor (Roche, Basel, Switzerland) for 10 sec on ice. After centrifugation (13,800 g, 4 °C, 10 min), the protein concentration of the supernatant was determined using the Bradford Protein Assay Kit (Bio-Rad). Samples were then heated at 95 °C in Laemmli sample buffer (Bio-Rad) and 2.5% mercaptoethanol, and 20-µg proteins were loaded per lane onto 4–20% Mini-PROTEAN TGX Precast gels (Bio-Rad). Following separation at 150 V, the proteins were transferred onto trans-blot turbo transfer pack membranes (Bio-Rad). The membranes were blocked using PVDF Blocking Reagent (Can Get Signal Kit, Toyobo, Osaka, Japan) for 1 h at room temperature. After 3 washes with Tris Buffered Saline with Tween 20 (TBST, Takara Bio), the membranes were incubated with antigen-specific antibodies (Supplemental Table S7) and diluted with Can Get Signal Solution 1 (Toyobo) at 4 °C overnight. Immunoreactive bands were visualized using a horseradish peroxidase conjugated secondary antibody (1:5,000, Santa-Cruz Biotechnology, Inc. Dallas, TX) diluted with Can Get Signal Solution 2 (Toyobo) and an enhanced chemiluminescence system (Thermo Fisher Scientific) using ChemiDoc MP (Bio-Rad). The relative expression level of each protein was normalized against α-tubulin (TUBA).

### Statistical analysis

Animal experiments and analyses were performed by different researchers blindly. Rats were randomly allocated to each group.

Continuous values are presented as the means ± standard error of the means (SEM). Statistical comparisons were made using a *t*-test for two-group comparisons, and analysis of variance (ANOVA) followed by Tukey’s post-hoc test for multiple between-group comparisons. Fisher’s exact test was used in the pathway analysis. A p < 0.05 was considered significant.

## Electronic supplementary material

Supplementary Infomation

## Data Availability

The data that support the findings of this study are available from the corresponding author upon reasonable request. Microarray data are available from GEO (GSE114031).
