# Supplementary material for: Pathophysiological and molecular mechanisms involved in renal congestion in a novel rat model
Source: Sci Rep. 2018 Nov 14;8:16808. doi: 10.1038/s41598-018-35162-4 (PMC6235885; doi:10.1038/s41598-018-35162-4)
Supplement: Supplementary file 1 — Supplementary Infomation [file 41598_2018_35162_MOESM1_ESM.pdf]

SUPPLEMENTAL INFORMATION

Title: Pathophysiological and molecular mechanisms involved in renal congestion in a novel rat model.

Authors and affiliations:

Satoshi Shimada, M.D.[1], Takuo Hirose, Ph.D.[1,2], Chika Takahashi, B.S.[2], Emiko Sato, Ph.D.[1,3], Satoshi Kinugasa, M.D., Ph.D.[1,2], Yusuke Ohsaki, Ph.D.[4], Kiyomi Kisu, B.S.[1], Hiroshi Sato, M.D., Ph.D.[1,3], Sadayoshi Ito, M.D., Ph.D.[1], and Takefumi Mori, M.D., Ph.D.[1,2]

[1]Division of Nephrology, Endocrinology and Vascular Medicine, Tohoku University Graduate School of Medicine, Sendai, Japan.

[2]Division of Nephrology and Endocrinology, Tohoku Medical and Pharmaceutical University, Sendai, Japan.

[3]Division of Clinical Pharmacology and Therapeutics, Tohoku University Graduate School of Pharmaceutical Sciences, Sendai, Japan.

[4]Division of Integrative Renal Replacement Therapy, Tohoku University Graduate School of Medicine, Sendai, Japan

Corresponding author: Takefumi Mori, M.D., Ph.D.

Division of Nephrology and Endocrinology,

Tohoku Medical and Pharmaceutical University

1-15-1, Fukumuro, Miyagino, 983-8536, Sendai, Japan.

Tel: +81–22–259–1221

Fax: +81–22–259–1232

E-mail: tmori@tohoku-mpu.ac.jp

Supplemental Figure S1

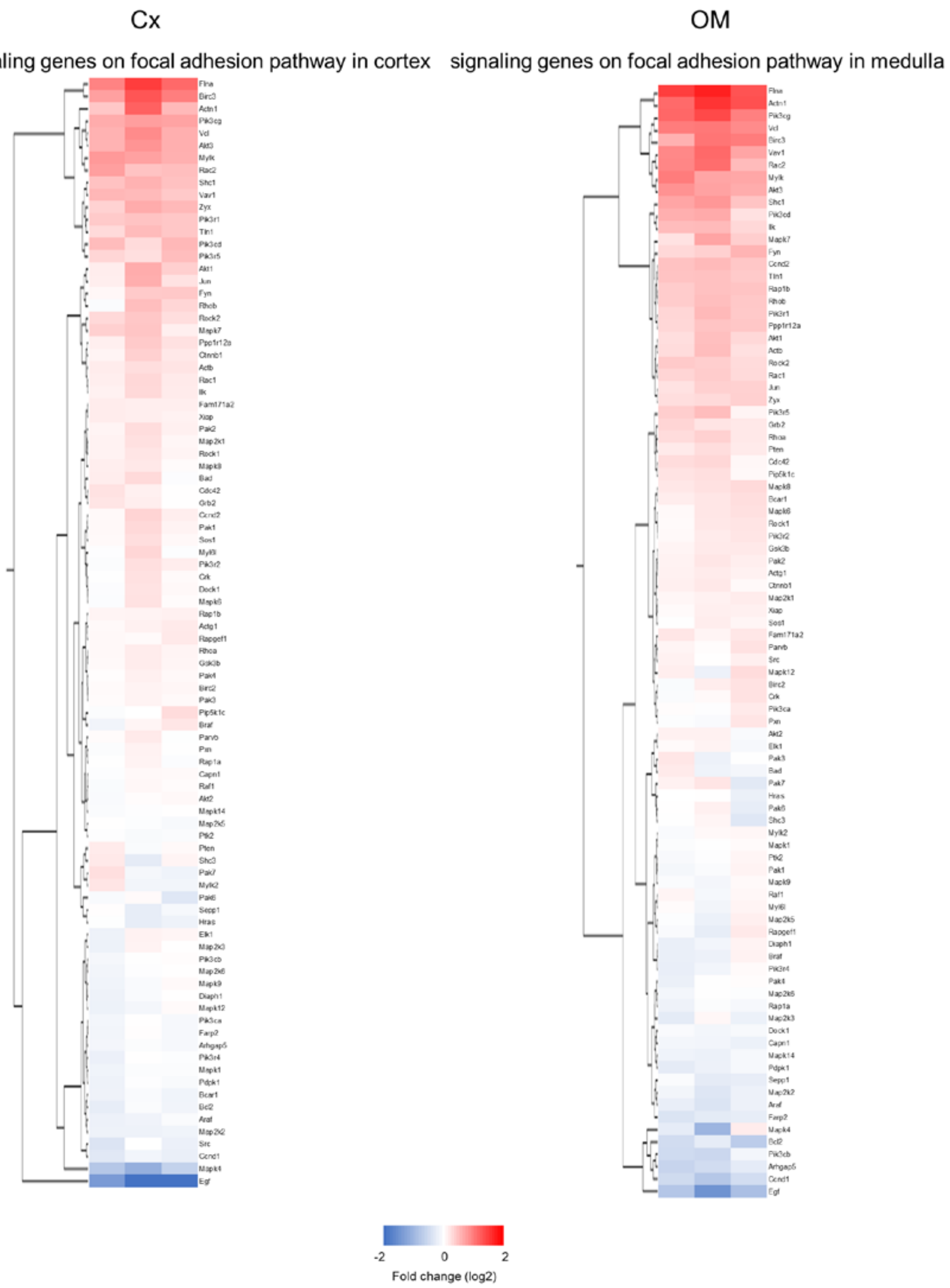

Supplemental Figure S1. Expression pattern of the focal adhesion pathway.

Signaling genes on the focal adhesion pathway were extracted from microarray results. Receptor and ligand genes on the focal adhesion pathway are shown in Figure 3. Congestive kidney expression level relative to the control kidney in logarithm is shown. Each line represents each gene (3 individuals). Cx: cortex; OM: outer medulla.

Supplemental Figure S2

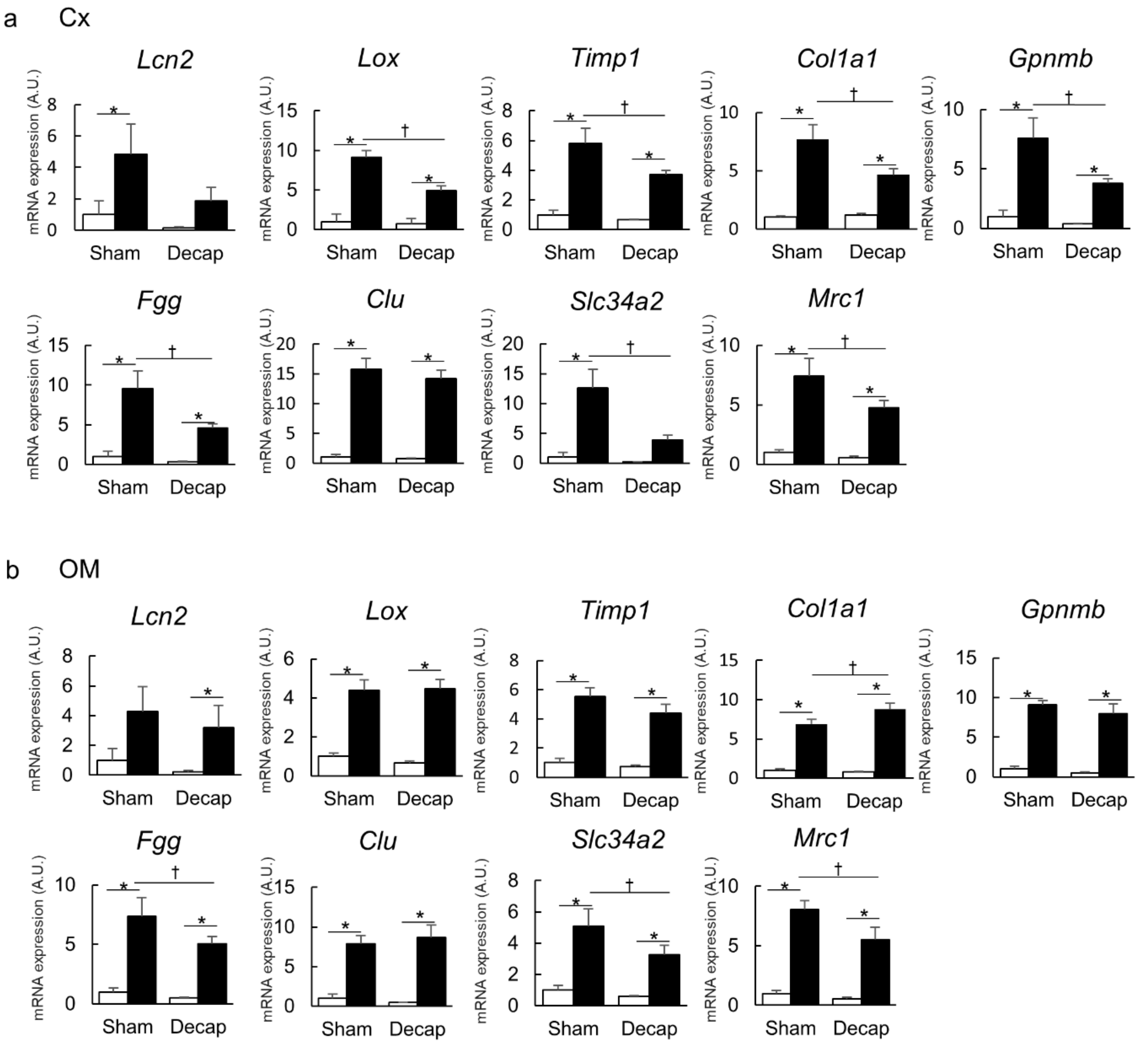

Supplemental Figure S2. qPCR of decapsulated IVC ligation model.

(a-b) qPCR of highly expressed genes in microarray analysis was performed for the left renal decapsulated IVC ligation model (Decap) and compared with sham IVC ligation model (Sham) in the cortex (a) and medulla (b). Sham n = 5, Decap n = 7. Data are presented as the means+SEM. *$p < 0.05$ versus the control kidneys, †$p < 0.05$ versus the sham-operated group; Tukey's test. □ indicates the control kidneys, ■ indicates the congestive kidneys. Cx: cortex; OM: outer medulla.

Supplemental Figure S3

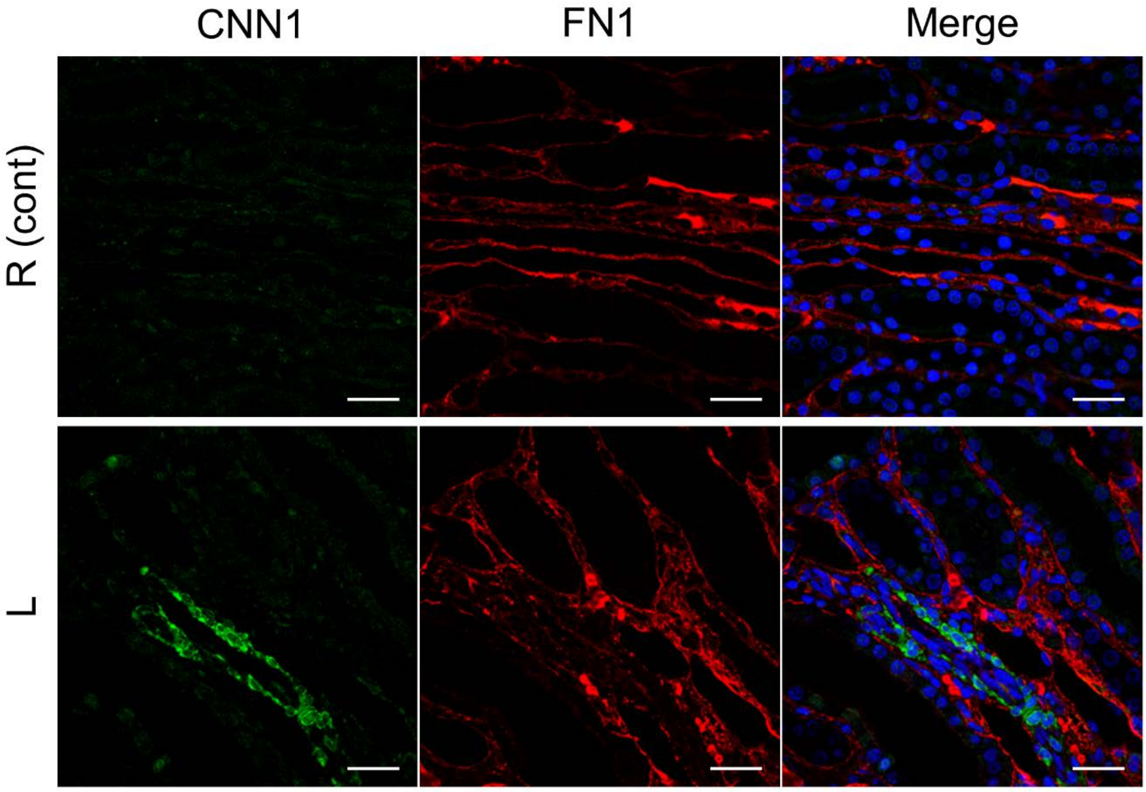

Supplemental Figure S3. Immunostaining of CNN1 and FN1.

CNN1 and FN1 costaining in the vasa recta. R (cont): control kidney; L: congestive kidney. CNN1: Calponin, FN1: Fibronectin 1. Scale bar, 20 μm.

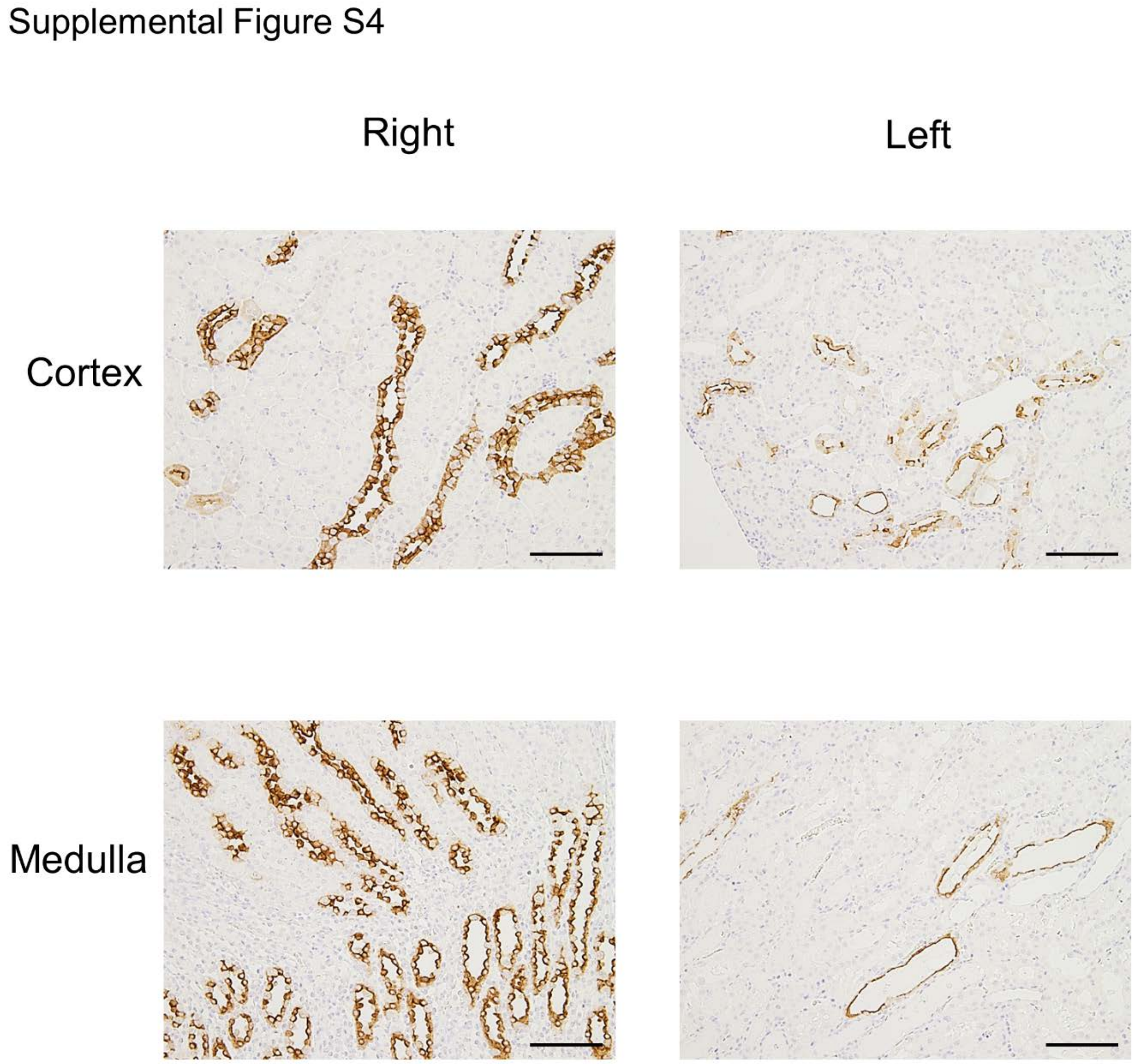

Supplemental Figure S4. Immunostaining of AQP2.

AQP2 staining in the cortex and medulla. Right: control kidney; Left: congestive kidney. Scale bar, 100 μm.

.

Supplemental Figure S5

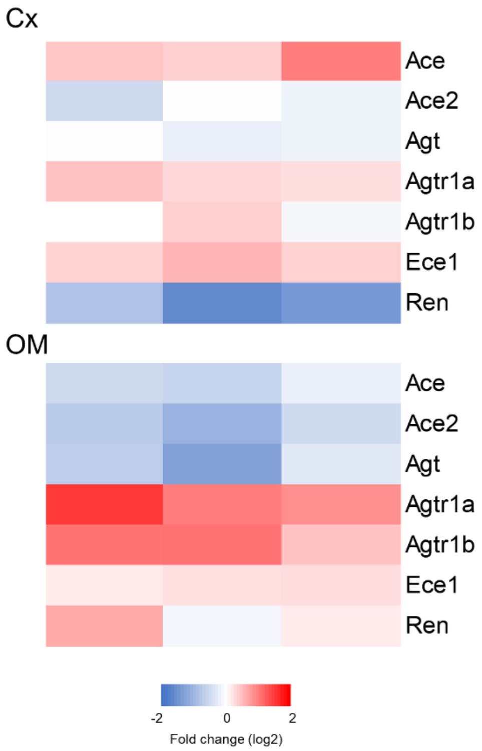

Supplemental Figure S5. Expression pattern of renin angiotensin aldosterone system. Renin angiotensin aldosterone system-related genes were extracted from microarray results. The congestive kidney expression level relative to the control kidney in logarithm is shown. Each line represents each gene. Each column represents each individual (n = 3). Cx: cortex; OM: outer medulla.

Supplemental Figure S6

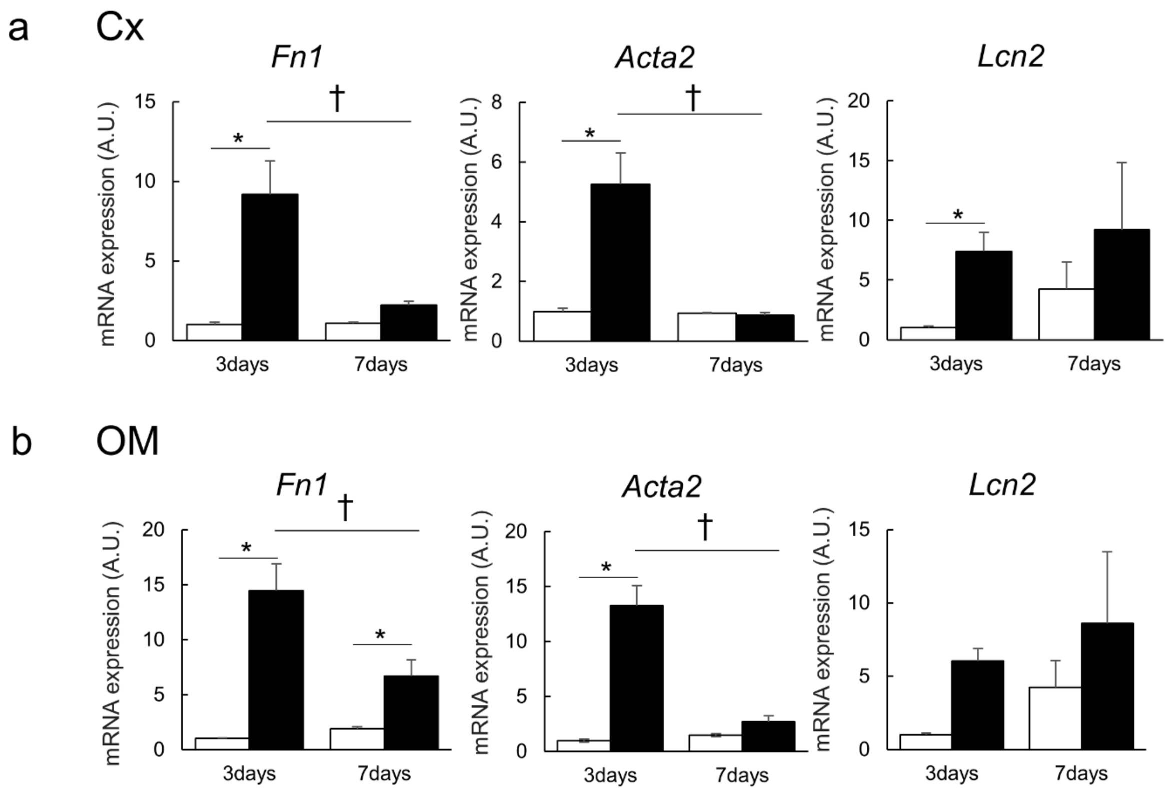

Supplemental Figure S6. qPCR of time course experiments.

(a-b) qPCR was performed for 7 days after IVC ligation and compared with result obtained 3 days after IVC ligation in the cortex (a) and medulla (b). n = 6. Data are presented as the means+SEM. *p < 0.05 versus the control kidneys, †p < 0.05 versus 3 days after operation; Tukey's test. □ indicates the control kidneys, ■ indicates the congestive kidneys. Cx: cortex; OM: outer medulla.

Supplemental Figure S7

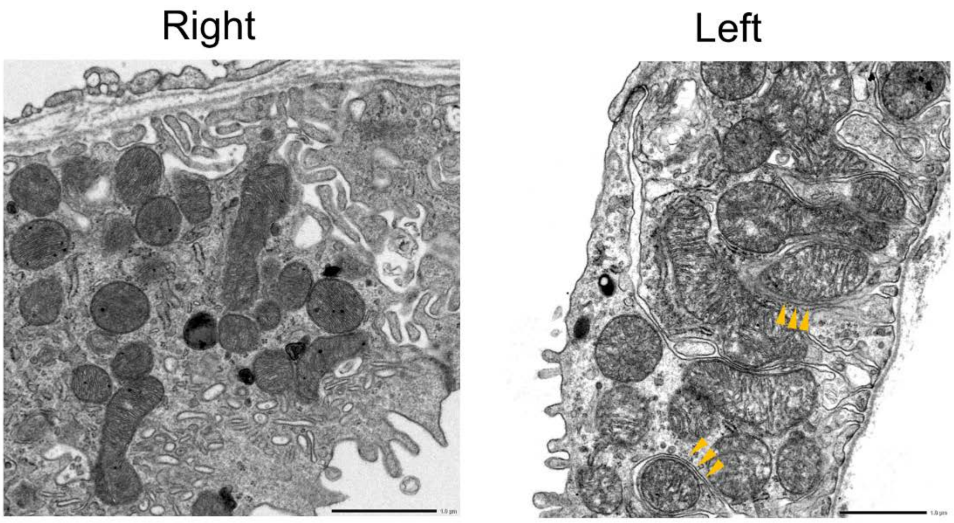

Supplemental Figure S7. Transmission Electron Microscopy on the collecting duct. Arrowheads indicate mitophagosomes. Right: control kidney; Left: congestive kidney. Scale bar, 1.0 μm.

Supplemental Figure S8

Fig. 4B

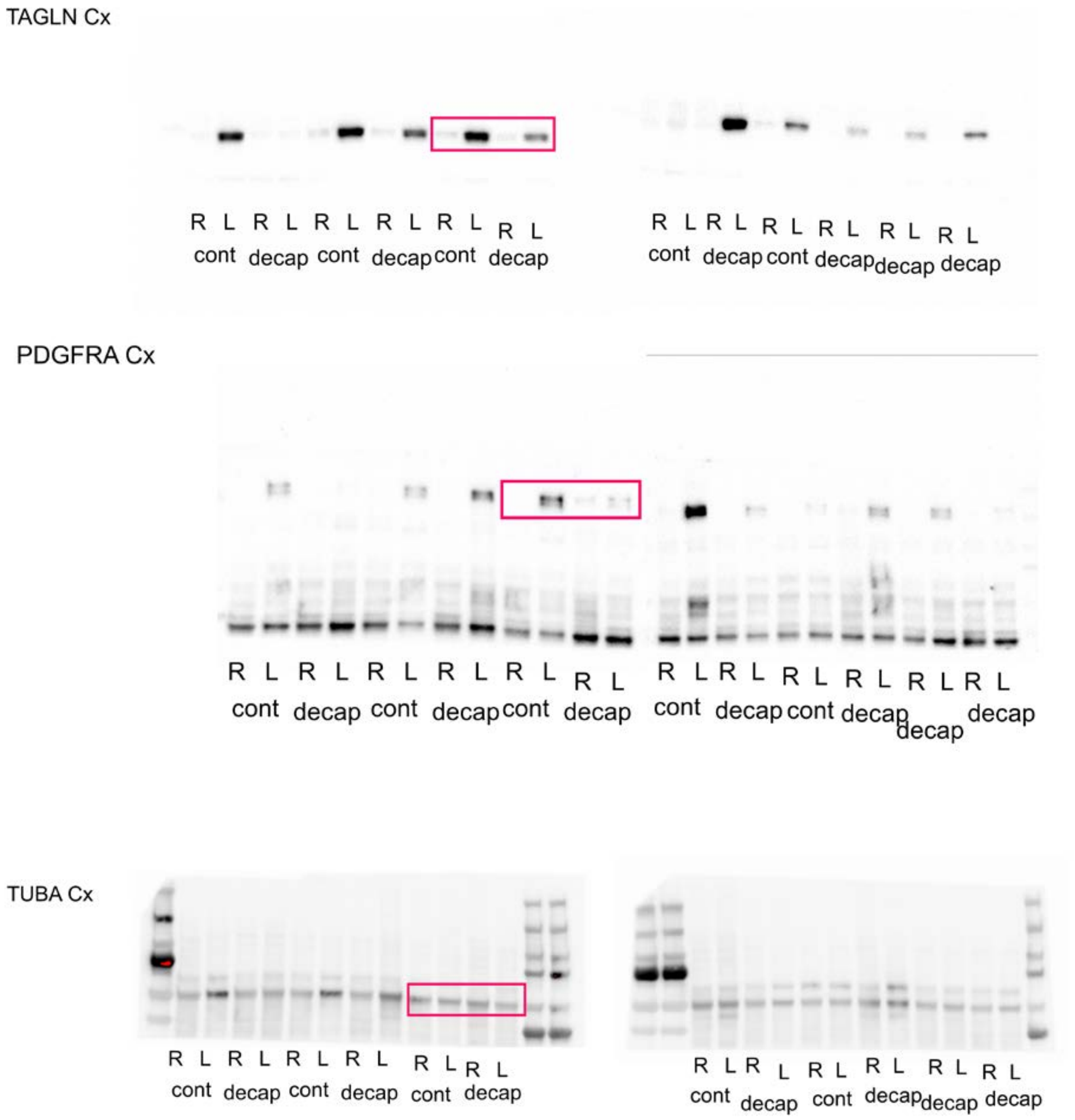

Supplemental Figure S8. Full-length blots of western blot analysis.

Supplemental Figure S8 (cont.)

Fig. 4B

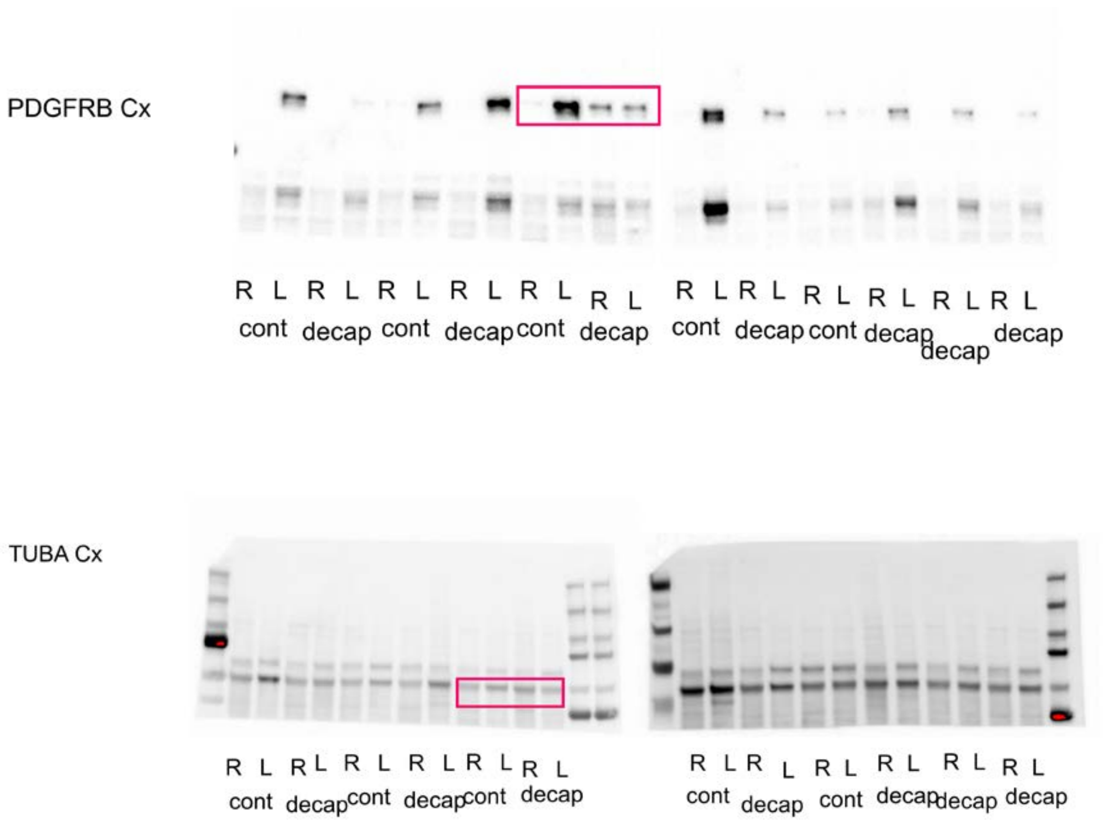

Supplemental Figure S8 (cont.)

Fig. 4B

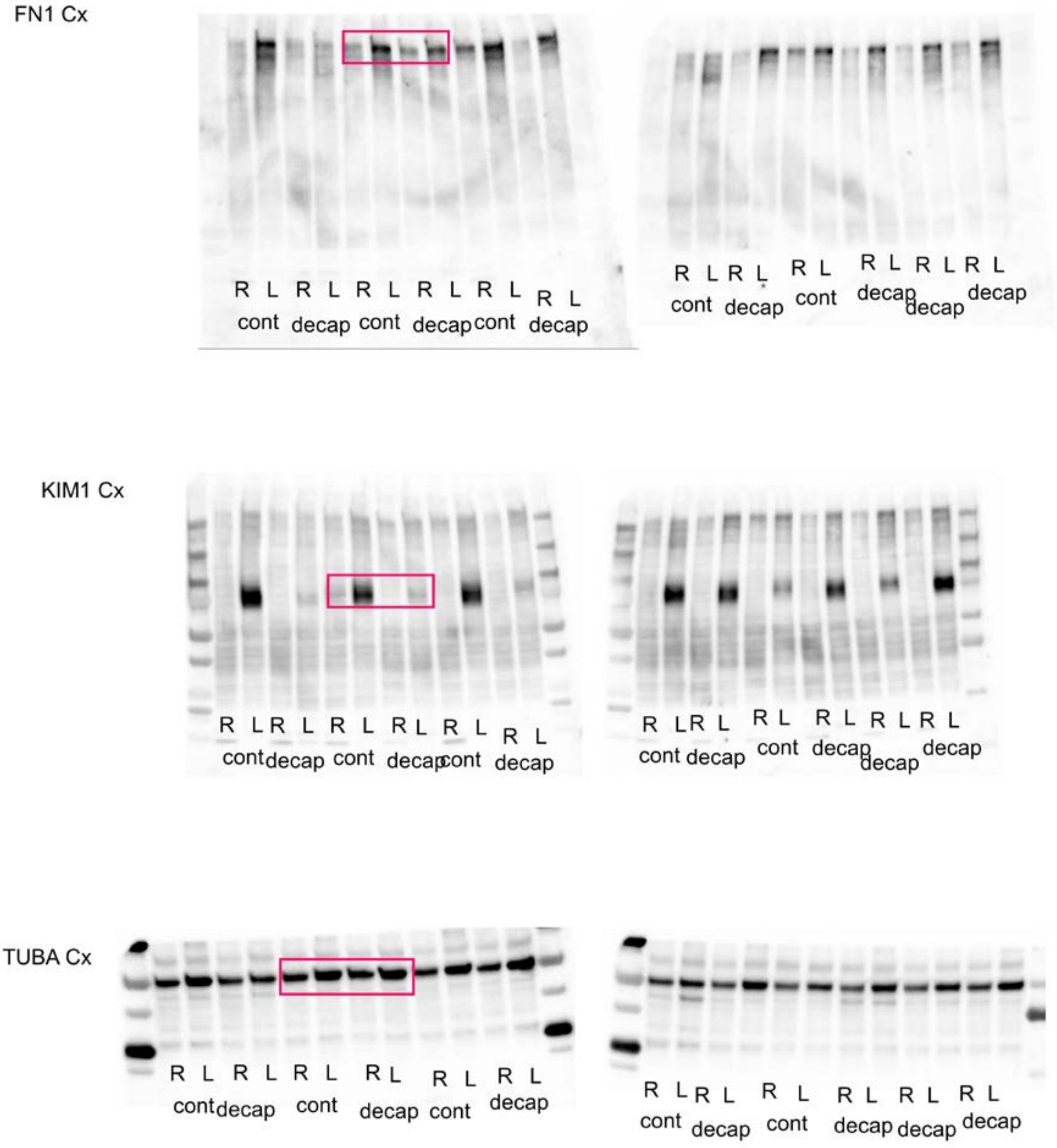

Supplemental Figure S8 (cont.)

Fig. 5B

TAGLNOM

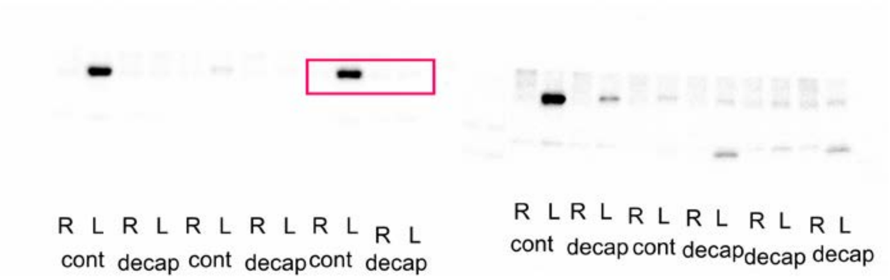

PDGFRA OM

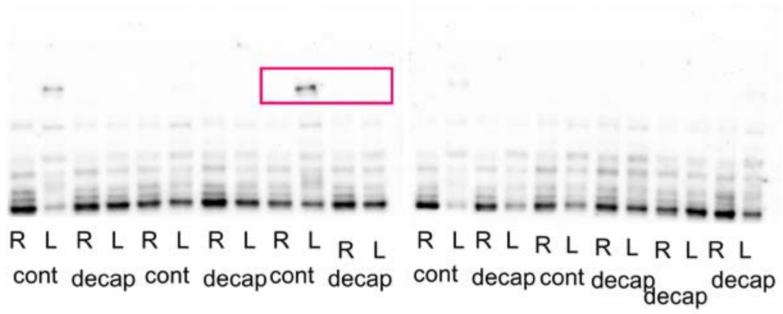

TUBA OM

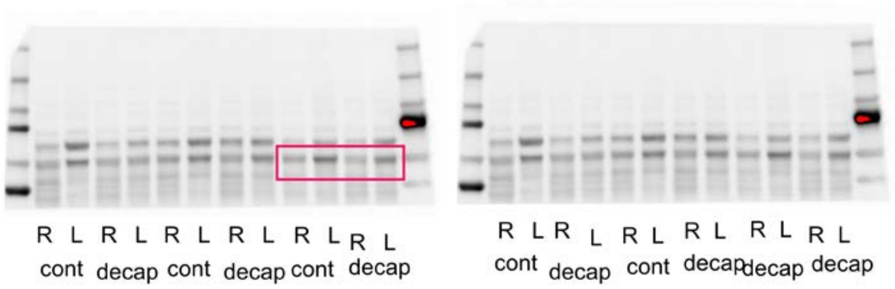

Supplemental Figure S8 (cont.)

Fig. 5B

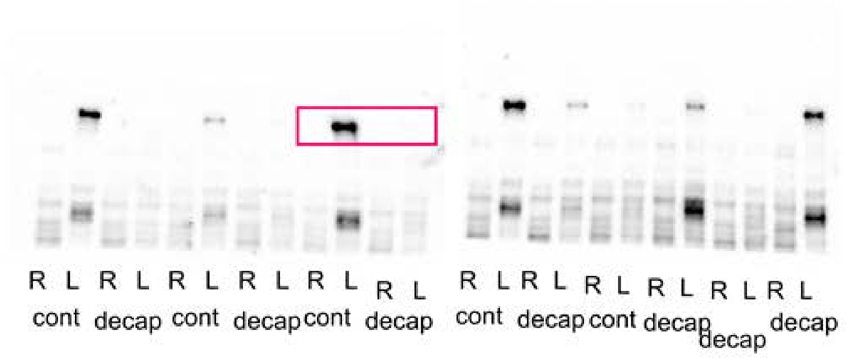

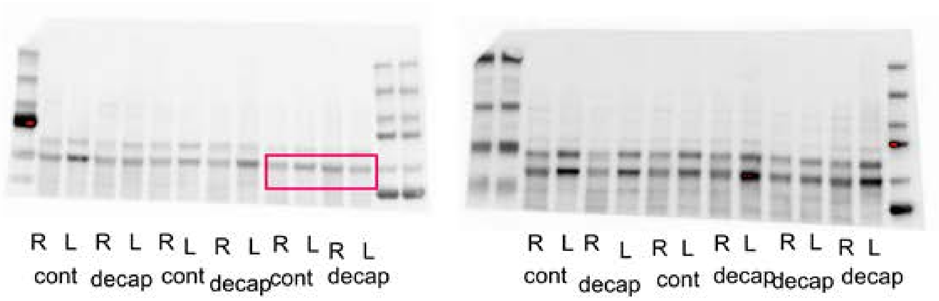

Supplemental Figure S8 (cont.)

Fig. 5B

FN1 OM

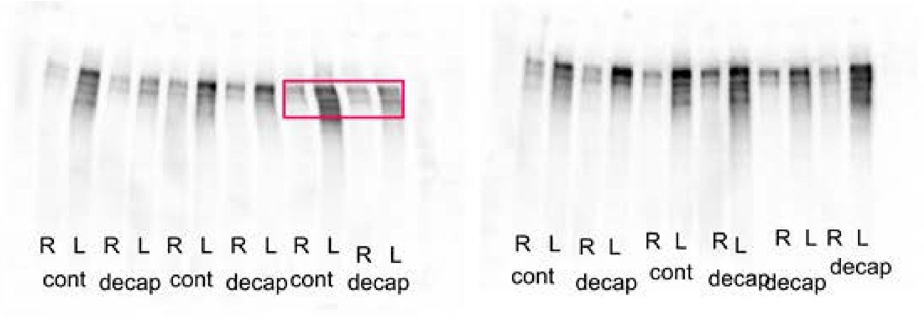

KIM1 OM

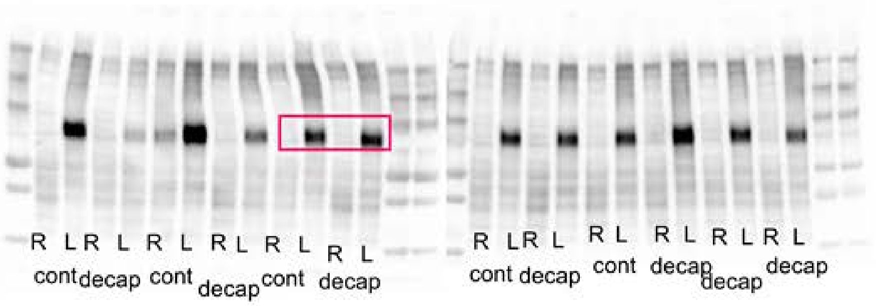

TUBA OM

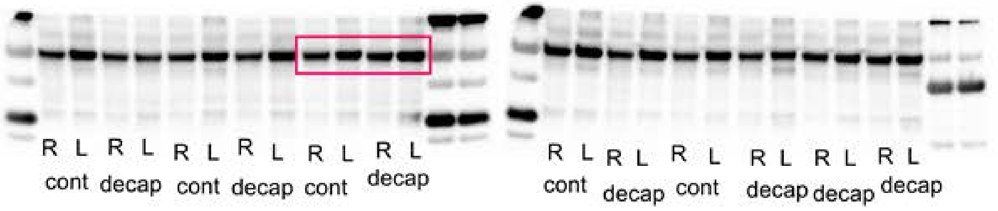

Supplemental Table S1. Biochemical analysis of chronic phase.

	sham		ligation	
Serum concentration				
TP (g/dL)	5.55±0.22		5.50±0.11	
Alb (g/dL)	3.33±0.11		3.25±0.04	
Cre (mg/dL)	0.25±0.01		0.33±0.02 *	
UN (mg/dL)	14.2±2.2		22.5±0.9 *	
Na (mEq/L)	144.6±1.7		143.1±1.4	
IP (mg/dL)	7.83±0.09		7.71±0.19	
	R	L	R	L
Urinary concentration				
UP (mg/dL)	101.0±17.1	110.4±20.4	183.5±31.7	157.4±37.1
Alb (µg/mL)	162.1±23.6	182.6±30.1	283.6±67.5	403.0±88.5 †
Cre (mg/dL)	44.3±13.5	41.6±8.6	75.6±17.7	33.0±6.8 †
UN (mg/dL)	1433±351	1408±311	2491±363	846±154 †
Na (mEq/L)	127±26.2	133.3±40.6	99.5±22.2	119±12.7
IP (mg/dL)	313.0±78.7	321.0±89.2	349.8±64.1	26.0±15.8 †‡
Urine cmponent total amount				
UP (µg/h)	596.9±98.2	618.8±146.9	710.0±91.0	296.4±34.1 †‡
Alb (µg/h)	97.2±14.8	103.3±23.4	104.5±15.9	78.1±8.3
Cre (µg/h)	212.1±31.6	192.4±18.2	292.1±43.7	62.8±8.0 †‡
UN (mg/h)	7.9±1.2	7.4±0.81	10.0±0.86	1.7±0.30 †‡
Na (µEq/h)	86.1±40.5	74.4±25.0	56.5±24.0	26.4±4.7
IP (µg/h)	1829±541	1652±337	1488±266	44.3±2.3 †‡
Creatinine correction				
UP (g/gCr)	2.70±0.48	2.89±0.67	2.67±0.51	5.22±0.97 †
Alb (g/gCr)	0.45±0.11	0.49±0.11	0.40±0.08	1.43±0.34 †‡

means±SEM. *p<0.05 versus sham operation group; by t-test. †p<0.05 versus control kidneys, ‡p<0.05 versus Sham group; by Tukey test. TP: total protein; Alb: Albumin; Cre: Creatinine; UN: Urine nitrogen; Na: sodium; IP: inorganic phosphorus; UP: Urinary protein.

Supplemental Table S2. Upregulated Gene Ontology terms in cortex.

GO ACCESSION	GO Term	p-value	corrected p-value	Count in	% Count in	Count in	% Count in Total
GO:0031012	extracellular matrix	5.51E-35	2.26E-30	48	21.2	380	2.0
GO:0005578	proteinaceous extracellular matrix	4.59E-30	9.42E-26	40	17.7	300	1.6
GO:0044420	extracellular matrix component	7.76E-24	1.06E-19	25	11.1	122	0.7
GO:0044421	extracellular region part	1.16E-22	1.19E-18	109	48.2	3628	19.5
GO:0005576	extracellular region	1.91E-21	1.57E-17	113	50.0	4003	21.5
GO:0005581	collagen trimer	5.00E-18	3.42E-14	17	7.5	69	0.4
GO:0005615	extracellular space	1.38E-17	8.08E-14	57	25.2	1322	7.1
GO:0043230	extracellular organelle	4.91E-16	2.52E-12	80	35.4	2619	14.1
GO:0030198	extracellular matrix organization	1.20E-15	5.49E-12	21	9.3	167	0.9
GO:0043062	extracellular structure organization	1.36E-15	5.59E-12	21	9.3	168	0.9
GO:1903561	extracellular vesicle	1.51E-15	5.63E-12	79	35.0	2614	14.0
GO:0005604	basement membrane	1.96E-15	6.69E-12	17	7.5	96	0.5
GO:0070062	extracellular exosome	3.93E-15	1.24E-11	78	34.5	2602	14.0
GO:0006950	response to stress	9.43E-15	2.76E-11	82	36.3	2875	15.4
GO:0031982<br>GO:0031988	vesicle	7.63E-14	2.09E-10	93	41.2	3661	19.7
GO:0009605	response to external stimulus	5.95E-13	1.53E-09	61	27.0	1897	10.2
GO:0005539	glycosaminoglycan binding	6.49E-13	1.57E-09	19	8.4	179	1.0
GO:0022610	biological adhesion	9.05E-13	2.06E-09	34	15.0	659	3.5
GO:0009611<br>GO:0002245	response to wounding	1.08E-12	2.34E-09	28	12.4	446	2.4
GO:0005201	extracellular matrix structural constituent	2.69E-12	5.53E-09	11	4.9	43	0.2

Supplemental Table S3. Downregulated Gene Ontology terms in cortex.

GO ACCESSION	GO Term	p-value	corrected p-value	Count in	% Count in	Count in	% Count in Total
GO:0002675	positive regulation of acute inflammatory response	1.82E-05	0.19	3	11.5	37	0.20
GO:0045907	positive regulation of vasoconstriction	3.52E-05	0.19	3	11.5	46	0.25
GO:0005615	extracellular space	4.60E-05	0.19	9	34.6	1322	7.10
GO:0002673	regulation of acute inflammatory response	9.51E-05	0.21	3	11.5	64	0.34
GO:0044421	extracellular region part	9.89E-05	0.21	14	53.8	3628	19.48
GO:0035413	positive regulation of catenin import into nucleus	1.02E-04	0.21	2	7.7	11	0.06
GO:0008217	regulation of blood pressure	1.29E-04	0.22	4	15.4	189	1.01
GO:0070011	peptidase activity, acting on L-amino acid peptides	1.49E-04	0.22	6	23.1	603	3.24
GO:0019229	regulation of vasoconstriction	1.59E-04	0.22	3	11.5	76	0.41
GO:0045177	apical part of cell	1.80E-04	0.22	5	19.2	390	2.09
GO:0008233	peptidase activity	2.01E-04	0.22	6	23.1	637	3.42
GO:1903524	positive regulation of blood circulation	2.06E-04	0.22	3	11.5	83	0.45
GO:0042307	positive regulation of protein import into nucleus	2.98E-04	0.26	3	11.5	94	0.50
GO:0005576	extracellular region	2.98E-04	0.26	14	53.8	4003	21.49
GO:1904591	positive regulation of protein import	3.26E-04	0.26	3	11.5	97	0.52
GO:0044057	regulation of system process	3.74E-04	0.26	5	19.2	457	2.45
GO:0003073	regulation of systemic arterial blood pressure	3.78E-04	0.26	3	11.5	102	0.55
GO:0004175<br>GO:0016809	endopeptidase activity	3.93E-04	0.26	5	19.2	462	2.48
GO:0050729	positive regulation of inflammatory response	4.01E-04	0.26	3	11.5	104	0.56
GO:0035412	regulation of catenin import into nucleus	4.66E-04	0.28	2	7.7	23	0.12

Supplemental Table S4. Upregulated Gene Ontology terms in medulla.

GO ACCESSION	GO Term	p-value	corrected p-value	Count in	% Count in	Count in	% Count in Total
GO:0031012	extracellular matrix	2.40E-44	1.31E-39	71	16.7	380	2.0
GO:0005578	proteinaceous extracellular matrix	6.09E-35	1.67E-30	56	13.2	300	1.6
GO:0005576	extracellular region	1.48E-32	2.03E-28	200	47.2	4003	21.5
GO:0044421	extracellular region part	1.13E-32	2.03E-28	189	44.6	3628	19.5
GO:0044420	extracellular matrix component	3.30E-25	3.62E-21	32	7.5	122	0.7
GO:0005615	extracellular space	8.63E-24	7.88E-20	94	22.2	1322	7.1
GO:0006950	response to stress	1.12E-22	8.78E-19	146	34.4	2875	15.4
GO:0005581	collagen trimer	1.77E-22	1.21E-18	24	5.7	69	0.4
GO:0009605	response to external stimulus	2.07E-22	1.26E-18	113	26.7	1897	10.2
GO:0009611<br>GO:0002245	response to wounding	1.21E-21	6.63E-18	51	12.0	446	2.4
GO:0006952<br>GO:0002217<br>GO:0042829	defense response	4.75E-20	2.37E-16	74	17.5	983	5.3
GO:0002376	immune system process	3.55E-19	1.62E-15	96	22.6	1594	8.6
GO:0022610	biological adhesion	6.43E-19	2.71E-15	58	13.7	659	3.5
GO:0044707	single-multicellular organism process	7.03E-19	2.75E-15	199	46.9	5036	27.0
GO:0048583	regulation of response to stimulus	1.17E-18	4.26E-15	144	34.0	3099	16.6
GO:0007155	cell adhesion	1.70E-18	5.73E-15	57	13.4	651	3.5
GO:0051239	regulation of multicellular organismal process	1.78E-18	5.73E-15	127	30.0	2561	13.8
GO:0009986<br>GO:0009928<br>GO:0009929	cell surface	5.51E-18	1.68E-14	62	14.6	780	4.2
GO:0002684	positive regulation of immune system process	1.02E-17	2.94E-14	58	13.7	699	3.8
GO:0048584	positive regulation of response to stimulus	1.28E-17	3.51E-14	96	22.6	1681	9.0

Supplemental Table S5. Downregulated Gene Ontology terms in medulla.

GO ACCESSION	GO Term	p-value	corrected p-value	Count in	% Count in	Count in	% Count in Total
GO:0055114	oxidation-reduction process	3.13E-08	8.06E-04	23	18.3	897	4.8
GO:0016491	oxidoreductase activity	1.08E-07	9.54E-04	20	15.9	735	3.9
GO:0044282	small molecule catabolic process	1.11E-07	9.54E-04	12	9.5	246	1.3
GO:0005215<br>GO:0005478	transporter activity	3.88E-07	1.37E-03	25	19.8	1199	6.4
GO:0015291<br>GO:0015290<br>GO:0015353<br>GO:0015404<br>GO:0015570	secondary active transmembrane transporter activity	5.11E-07	1.37E-03	11	8.7	232	1.2
GO:0015711	organic anion transport	5.15E-07	1.37E-03	13	10.3	339	1.8
GO:0015849	organic acid transport	5.34E-07	1.37E-03	11	8.7	233	1.3
GO:0022892	substrate-specific transporter activity	4.08E-07	1.37E-03	23	18.3	1035	5.6
GO:0044710	single-organism metabolic process	3.36E-07	1.37E-03	42	33.3	2845	15.3
GO:0046942	carboxylic acid transport	4.90E-07	1.37E-03	11	8.7	231	1.2
GO:0006811	ion transport	8.32E-07	1.95E-03	24	19.0	1163	6.2
GO:0022891	substrate-specific transmembrane transporter activity	1.36E-06	2.92E-03	20	15.9	863	4.6
GO:0022857<br>GO:0005386<br>GO:0015646	transmembrane transporter activity	1.50E-06	2.97E-03	21	16.7	949	5.1
GO:0015075	ion transmembrane transporter activity	1.80E-06	3.09E-03	19	15.1	800	4.3
GO:0015297<br>GO:0015300	antiporter activity	1.75E-06	3.09E-03	7	5.6	85	0.5
GO:0006820<br>GO:0006822	anion transport	2.05E-06	3.30E-03	14	11.1	447	2.4
GO:0016054	organic acid catabolic process	2.47E-06	3.34E-03	9	7.1	171	0.9
GO:0043436	oxoacid metabolic process	2.24E-06	3.34E-03	19	15.1	812	4.4
GO:0046395	carboxylic acid catabolic process	2.47E-06	3.34E-03	9	7.1	171	0.9

Supplemental Table S6. Primer information.

Symbol	primer set ID
Acta2 (α-SMA)	RA060203
Clu	RA047380
Col1a1	RA065609
Col1a2	RA069679
Col3a1	RA063380
Des	RA058597
Fgg	RA045154
Fn1	RA055827
Gapdh	RA015380
Gpnmb	RA059101
Havcr1 (Kim1)	RA057664
Lcn2 (Ngal)	RA046649
Lox	RA047248
Mmp2	RA068619
Mrc1	RA071208
Pdgfra	RA059727
Pdgfrb	RA048695
Slc34a2	RA062011
Spp1 (Opn)	RA017345
Tagln (SM22)	RA063024
Timp1	RA015383
Vim	RA050877

Supplemental Table S7. Antibody information.

Antigen	Company	Catalog number	Host	WB	IHC/IF
ACTA2 (α-SMA)	Dako	M0851	mouse		1: 200
AQP2	Sigma-Aldrich	A7310	rabbit		1: 3000 *
CNN1	Sigma-Aldrich	C2687	mouse		1: 1000 *
DES	Abcam	ab15200	rabbit		1: 200 †
FN1	Merck	F3648	rabbit	1: 1000	1: 400 *
KIM1	R&D system	AF3689	goat	1: 1000	1: 1250 *
NPHS1	PROGEN	GP-N2	guinea pig		1: 200 †
OPN	Santa Cruz	sc-10591	goat		1: 50
PDGFRA	Cell Signaling	3174s	rabbit	1: 1000	1: 500 *
PDGFRB	Abcam	ab32570	rabbit	1: 1000	1: 100 *
Pimonidazole	Hypoxyprobe	HP1-100	mouse		1: 50 *
TAGLN (SM22)	Abcam	ab14106	rabbit	1: 1000	1: 200 *
TUBA	Cell Signaling	3873	mouse	1: 1000	
VIM	Abcam	ab92547	rabbit		1: 1250 *

* The antigens which were retrieved by microwave heating for 5 min in 10 mmol/L citrate buffer.

† The antigens which were retrieved by microwave heating 5 min in 5 mmol/L ethylenediaminetetraacetic acid buffer.
